# Deep Learning for Accurate Diagnosis of Liver Tumor Based on Magnetic Resonance Imaging and Clinical Data

**DOI:** 10.3389/fonc.2020.00680

**Published:** 2020-05-28

**Authors:** Shi-hui Zhen, Ming Cheng, Yu-bo Tao, Yi-fan Wang, Sarun Juengpanich, Zhi-yu Jiang, Yan-kai Jiang, Yu-yu Yan, Wei Lu, Jie-min Lue, Jia-hong Qian, Zhong-yu Wu, Ji-hong Sun, Hai Lin, Xiu-jun Cai

**Affiliations:** ^1^Department of General Surgery, School of Medicine, Sir Run Run Shaw Hospital, Zhejiang University, Hangzhou, China; ^2^State Key Laboratory of CAD&CG, Zhejiang University, Hangzhou, China; ^3^Department of Radiology, School of Medicine, Sir Run Run Shaw Hospital, Zhejiang University, Hangzhou, China; ^4^Department of Medical Imaging, Hwa Mei Hospital, University of Chinese Academy of Sciences, Ningbo, China; ^5^Department of Surgical Oncology, School of Medicine, Sir Run Run Shaw Hospital, Zhejiang University, Hangzhou, China

**Keywords:** liver cancer, liver mass, deep learning, diagnosis, artificial intelligence, MRI

## Abstract

**Background:** Early-stage diagnosis and treatment can improve survival rates of liver cancer patients. Dynamic contrast-enhanced MRI provides the most comprehensive information for differential diagnosis of liver tumors. However, MRI diagnosis is affected by subjective experience, so deep learning may supply a new diagnostic strategy. We used convolutional neural networks (CNNs) to develop a deep learning system (DLS) to classify liver tumors based on enhanced MR images, unenhanced MR images, and clinical data including text and laboratory test results.

**Methods:** Using data from 1,210 patients with liver tumors (*N* = 31,608 images), we trained CNNs to get seven-way classifiers, binary classifiers, and three-way malignancy-classifiers (Model A-Model G). Models were validated in an external independent extended cohort of 201 patients (*N* = 6,816 images). The area under receiver operating characteristic (ROC) curve (AUC) were compared across different models. We also compared the sensitivity and specificity of models with the performance of three experienced radiologists.

**Results:** Deep learning achieves a performance on par with three experienced radiologists on classifying liver tumors in seven categories. Using only unenhanced images, CNN performs well in distinguishing malignant from benign liver tumors (AUC, 0.946; 95% CI 0.914–0.979 vs. 0.951; 0.919–0.982, *P* = 0.664). New CNN combining unenhanced images with clinical data greatly improved the performance of classifying malignancies as hepatocellular carcinoma (AUC, 0.985; 95% CI 0.960–1.000), metastatic tumors (0.998; 0.989–1.000), and other primary malignancies (0.963; 0.896–1.000), and the agreement with pathology was 91.9%.These models mined diagnostic information in unenhanced images and clinical data by deep-neural-network, which were different to previous methods that utilized enhanced images. The sensitivity and specificity of almost every category in these models reached the same high level compared to three experienced radiologists.

**Conclusion:** Trained with data in various acquisition conditions, DLS that integrated these models could be used as an accurate and time-saving assisted-diagnostic strategy for liver tumors in clinical settings, even in the absence of contrast agents. DLS therefore has the potential to avoid contrast-related side effects and reduce economic costs associated with current standard MRI inspection practices for liver tumor patients.

## Introduction

Liver cancer is the second leading cause of cancer-related deaths worldwide (1) and the incidence rate has been growing on a global scale (2), which is in contrast to the stable incidence or declining trends for most cancers (3). Hepatocellular carcinoma (HCC) accounts for 90% of primary liver cancers, and could result in a major global health problem. Early-stage HCC detection and diagnosis can allow the patients to receive the treatment earlier and achieve better survival rates (1). All the continental and national liver disease societies have recommended that surveillance should be carried out for high- risk patients with cirrhosis (4). Ultrasonography as the preferred test for surveillance is unsatisfactory because of the limitations of ultrasound operator dependency and its low sensitivity to small liver cancers (5). Dynamic contrast-enhanced imaging is recommended as the first-line diagnostic tool for HCC when the screening test result is abnormal (6). Undoubtedly, compared with computed tomography (CT), enhanced MRI is the better choice because of its various tissue contrast mechanisms and it being only way to assess all major and auxiliary imaging features (7). However, enhanced MRI could not be used widely like ultrasonography in screening and surveillance owing to its high cost and the risk of contrast- related side effects (8–12).

Actually, even with enhanced MRI, it still remains challenging to diagnose, owing to liver tumor diversity and complex imaging features. In addition to HCC, primary malignant tumors in the liver include intrahepatic cholangiocarcinoma (ICC), mixed hepatocellular-cholangiocarcinoma (HCC-CC), and other rare tumors (13, 14). The liver is also the target of metastasis for many malignant tumors, such as colorectal, pancreatic, neuroendocrine, breast cancer, etc. Moreover, there are several types of benign tumors in the liver, such as cyst, hemangioma, focal nodular hyperplasia (FNH), adenomas, high-risk cirrhotic nodules [regenerative nodules (RN), and dysplastic nodules (DN)] (14). The evaluations of images are generally subjective and are possibly affected by radiologists' experience (15, 16).

Unlike traditional image-dependent “semantic” features evaluation from human experts, deep learning can automatically learn feature representations from sample images with convolutional neural networks (CNNs) (17, 18). It has shown to match or even surpass human performance in the application of specific tasks and may even discover additional differential features not yet identified in current radiological practice (19). CNNs have been achieving good performances in medical imaging for several tumor types (20–22), but for liver tumors only a few exploratory studies have been reported (16, 23–25). These studies trained models based on enhanced images from contrast-enhanced CT/MRI in small-scale (<500 patients) datasets, however, they only considered specific liver tumor categories and did not simulate clinical practice conditions, which restricted their utility in the diagnostic decision-making phases of the workflow. In addition, the potential diagnostic value of clinical information and unenhanced sequences, including T1 pre-contrast, T2, and diffusion sequences, were not evaluated in deep-learning models. Here, we report the results of a large study of liver tumors, which covered all types of hepatic local lesions except for inflammatory masses. There were two aims of this study: First, we developed CNNs that implemented assisted diagnosis for liver tumors by classifying them in seven categories. Second, we developed a CNN that utilized unenhanced sequences to distinguish malignant from benign tumors, then, our modified CNN that combines unenhanced images with clinical data achieved end-to-end output for precise classification of malignant tumors. These models were integrated into the DLS. In an external independent cohort, their performance was compared with experienced radiologists that had read all sequences and clinical information.

## Materials and Methods

### Patients

This study was approved by the independent institutional review boards of Sir Run Run Shaw Hospital and performed according to the Helsinki declaration. In this study, a sample size was not prespecified. Instead, we included the largest possible number of patients with liver tumors to ensure that deep learning models were trained as fully as possible. The inclusion criteria were as follows: (1) participants had liver tumors, and (2) participants accepted enhanced MRI inspection. The exclusion criteria were as follows: (1) those who had accepted treatment related to the lesion before MRI inspection, including surgery, transcatheter arterial chemoembolization (TACE), radiofrequency ablation, chemotherapy, radiotherapy, targeted drug therapy, etc.; (2) those with inflammatory lesions; (3) those with a clinically diagnosed malignancy (without pathology confirmed); (4) any missing important medical records or laboratory results of the malignancy individuals; and (5) unqualified image quality. Only patients who had malignancies confirmed by biopsy or post-surgical histopathology were enrolled. The diagnosis of some benign lesions was supported by histopathology, but the labels of other benign lesions without surgery provided by the imaging diagnosis report were considered as our gold standard. For these liver masses diagnosed with a combination of clinical information and imaging criteria, the follow-up time was 6–10 months. Those labels were the result of a consensus as explained by the radiology department: The report was firstly given by the doctor who read the images. Then, the report was reviewed by the senior doctor. In case of a disagreement, the final decision was confirmed by a department conference. The test set consists of liver focal lesions enhanced by MRI in the same hospital between July 2018 to December 2018 according to the include and exclude criteria above (Figure S1). In addition, general demographics for all patients, pathology reports (if any), and malignant patients' related medical history, liver-related symptoms, and laboratory test results were documented.

### Taxonomy

Three groups of models were trained. The first task was to classify the liver focal lesions of the training set into seven categories: 0. cyst, 1. hemangioma, 2. focal liver lesion (FNH), 3. other benign nodules (cirrhotic nodules, RN, DN, rare benign tumors), 4. HCC, 5. metastatic malignant tumors from other sites (colorectal, breast, lung, pancreas, etc.), 6. primary hepatic malignancy other than HCC (intrahepatic cholangiocarcinoma, adenocarcinoma, etc.). The second task was to divide all the tumors into two categories: a. benign (include 0, 1, 2, 3 above), b. malignant (include 4, 5, and 6). In the third task, malignant tumors were divided into three categories the same as category 4, 5, and 6 in the seven-category method above. The test set was also classified as described above and labeled. Those classification models included all liver mass-like lesions (except for inflammatory lesions). In category 3, 5, and 6, several different specific tumor types were all mixed in the training and test set. Three experienced radiologists independently read and labeled the MRI of the validation set following this standard and they could refer to additional information such as medical history, laboratory test results, etc. according to daily clinical work habits.

### MRI Acquisition Protocol

Abdominal MRIs were performed in a supine position with 1.5-T, 3-T, and 750 W MR scanners, including GE MR Singna HDX 3.0T, GE MR Singna HD Excite scanners 1.5T, Simen MR Skyra 3.0T, Simen magnetom avanto Dot1.5T, and GE discovery MR 750 scanners. T2-weighted sequence, diffusion-weighted sequence (b-values: 800 s/mm^2^) from standard institutional liver MR imaging protocols were performed with acquisition times of 2–2.5 and 2–2.5 min. Contrast-enhanced T1 sequences were used with acquisition times of 12–18 s. Two different contrast agents were used, i.e., Gadopentetate dimeglumine and Gadoxetic Acid Disodium (Primovist) at doses of 0.2 and 0.1 mmol/kg, respectively. Except for pre-contrast T1, T2, and diffusion images, post- contrast images were analyzed, including late arterial phase (~15 s post-injection), portal venous phase (~60 s post-injection), and equilibrium phase (~3 min post- injection). Imaging parameters varied across different scanners and time frames (Table S4).

### Image Processing

Eligible MRI images were downloaded from the Picture Archiving and Communication Systems (PACS) and stored as Digital Imaging and Communications in Medicine (DICOM) files. Six sequences were selected and the region of interest (ROI) on T2 sequence was annotated. All the sequences were resampled to a resolution of 0.7 × 0.7 × 7 mm. Then the annotations of the other five sequences were generated according to the origin and spacing information of sequences, which were checked manually. Software was developed to correct cases that were not matched. SimpleITK was used to read DICOM images. After preprocessing, resampling, and configuration, the DICOM files were converted to images in preparation for the training. Some common augmentation methods were performed on the images such as rotation, flipping, scaling, shifting, and shearing.

### Deep Learning Model Development

Deep CNNs have achieved good results in medical image classification in recent years. The most straightforward way to improve the performance of deep CNNs is to increase their depth and width. Szegedy et al. (26) proposed a deep CNN architecture codenamed Inception that increased the width of each stage. Multiple versions of Inception-Net have been widely used in classification tasks. Residual connections introduced by He et al. (27) make it easy to train very deep networks. Inception- ResNet, which combines both ideas, outperforms the previous networks. We utilized a Google Inception-ResNet V2 CNN architecture (28) that was pre- trained on approximately 1.28 million images (1,000 object categories) from the 2014 ImageNet Large Scale Visual Recognition Challenge (29), then we removed the final classification layer from the network and retrained it with our dataset, fine-tuning the parameters across all layers (30). Our CNN was trained using backpropagation. The loss function was defined as categorical cross entropy between predicted probability and the true class labels in multi-class classification problems. We used stochastic gradient descent (SGD) optimization, with the same global learning rate of 0.1, a decay factor of 0.5 every 20 epochs, a momentum of 0.9, and batch size of 16 for training the weights. The TensorFlow (31) deep learning framework was used to train, validate, and test our network. We resized each image to 299 × 299 pixels in order to make it compatible with the original dimensions of the Inception-ResNet V2 network architecture. During training, images were augmented. Each image was rotated randomly between −40° and 40° and flipped vertically and horizontally with a probability of 0.5. Five-fold cross-validation was used in training CNN, and the parameters of the model with the highest average accuracy on the cross-validation sets were used to train CNN on the whole training set so as to get the final model.

For our dataset, either six (T1, T2 diffusion, late arterial, portal venous, and equilibrium) or three (T1, T2 and diffusion) sequences and clinical data were applied as inputs of our model. Each group of images from six or three sequences can be input to the network through different channels. For the three-way classification model, we modified the network to receive the image and clinical data as inputs simultaneously. The convolution layers were used to extract features from images, and then these features together with encoded clinical data were input to the fully connected layer for classifying liver tumors. Our deep learning model can accept clinical data as input. Clinical data was encoded using one-hot encoding. For example, gender can be encoded as [0,1] for male and [1,0] for female. The output of network is a one-dimensional vector about the predicted value for each group of images, which is expressed as [P1, P2,…, Pi], i represents i-category classification, Pi represents the predicted value for the i-th category. To calculate the individual-level predicted value, the predicted vector for each image-group of one patient was summed up, then the category with the largest value was used as the final diagnosis of this patient.

### Statistical Analysis

Upon finishing the training phase, the performance was evaluated using the validation set, which is composed of images from patients of an external independent dataset not used during the training. Then the predicted probability of each patient was obtained by aggregating the predicted probability values of each group of images. For classification purposes, the ROC curve was used to show the diagnostic ability of the deep learning model in discriminating specific class from others. The ROC curve and the corresponding area under ROC curve (AUC) for each class were calculated in each model using the python library sklearn (32). Differences between various AUCs were compared by using a Delong test. Average sensitivity and specificity of radiologists were displayed in ROC charts, then the sensitivity and specificity of radiologists' consensus were used to compare with the models (see in Tables). Ninety-five percentage CIs for sensitivity, specificity, and accuracy were calculated with the Clopped-Pearson method (33). *P*-values for sensitivity and specificity comparisons were computed using McNemar's test (binomial distribution for exact probabilistic method). In addition, the agreements between the predicted results with pathological/formal report were compared using the Cohen's Kappa statistic (34, 35) and *P*-values were estimated by a two-sample two-tailed z-test score. All tests were two-tailed, and *p* ≤ 0.05 was considered to be indicative of statistical significance.

Confusion matrixes demonstrated the mis-classification similarity between the CNNs and human experts. Element (i, j) of each confusion matrix represented the empirical probability to predict class j given that the ground truth was class i.

To analyze the internal features learned by the CNNs of validation sets, the Barnes- Hit implementation of the t-SNE technique (t-distributed Stochastic Neighbor Embedding) (36) was used to reduce the dimensionality and facilitate the visualization of the classes. The values associated with the last fully hidden connected layer were used as an input, and theta was set to 0.5, perplexity to 50, and 10,000 iterations. Colored point clouds represented the different disease categories, representing how the algorithm clusters the diseases.

### Saliency Map

To gain further intuition into how the network made its decisions, saliency maps (37), that can visualize the pixels that a network is fixating on for its prediction, are increasingly being used (20, 38). Back-propagation is an application of the chain rule of calculus to compute loss gradients for all weights in the network. The loss gradient can also be back-propagated to the input data layer. By taking the L1 norm of this input layer loss gradient across the RGB channels, the resulting heat map intuitively represents the importance of each pixel for diagnosis. We generated saliency maps about seven categories and typical individual examples to visualize areas on the images that were deemed important for the classification results. All saliency maps were produced using Keras 2.2.0.

## Results

### Training and Validation Cohort

Between January 1, 2014 to June 30, 2018, MRI images were obtained for the training set from the hepatic focal lesions database in Sir Run Run Shaw Hospital affiliated to Medicine School, Zhejiang University. According to the inclusion and exclusion criteria (Figure S1), the complete training set consisted of 31608 MRI images from 1,210 individuals, including 5,268 groups, and each group included six images from different scan sequences (T2, diffusion, Pre-contrast T1, late arterial, portal venous, equilibrium phase). Between July 1, 2018 to December 31, 2018, 6,816 images from 201 individuals for the validation set were obtained from Sir Run Run Shaw Hospital according to strict enrollment criteria to minimize selective bias (Figure S1), which ensured that the validation set could reflect the disease composition and distribution waiting to be diagnosed in real-world clinical scenarios. Only malignancies that had been confirmed by biopsy or post-surgical histopathology were enrolled. The diagnosis of some benign lesions was supported by histopathology, but the labels of other benign lesions without surgery provided by formal imaging diagnosis reports were considered as our gold standard. The study classified hepatic local lesions into seven categories: 0. cyst, 1. hemangioma, 2. focal liver lesion (FNH), 3. other benign nodules [cirrhotic nodules, regenerative nodules(RN), dysplastic nodules(DN), rare benign tumors], 4. HCC, 5. metastatic malignant tumors from other sites (colorectal, breast, lung, pancreas, etc.), and 6. primary hepatic malignancy other than HCC (adenocarcinoma, Intrahepatic cholangiocarcinoma, etc.). 0, 1, 2, and 3 above belonged to benign, 4, 5, and 6 belonged to malignant (see Methods, Taxonomy in details). Baseline characteristics of the training set and validation set are shown in Table S1. The disease composition and distribution of the validation set were not exactly the same as the training set (Table S2). According to TRIPOD statement (39), this validation set can be regarded as an external independent set. The training dataset in the current study is the largest published liver-enhanced MRI dataset with the most types of liver tumors (non- inflammatory lesion).

### Deep-Learning Frameworks for Liver Tumor Classification

**Figure 1:**
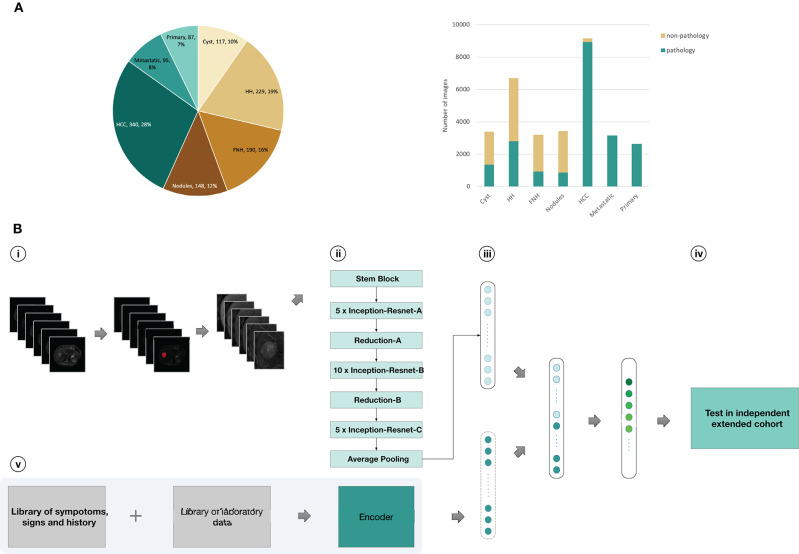
Data and strategy. **(A)**, Number of patients and images per category. **(B)**, Strategy for development and validation. (**B**, i), Magnetic resonance images of patients in training set were first downloaded from the PACS; Liver tumors was outlined in related images of T2-weighted sequences as Regions of interest (ROI) by ITK-SNAP software; pre-processed images and obtained six different scan sequences pictures for each cross section of the lesion (T2, diffusion,T1 pre-contrast, late arterial phase, portal venous phase, equilibrium phase); (**B**, ii), six sequences of each cross section input to CNN as a whole-image from six channels, encoded clinical data was input to CNN; (**B**, iii), the Inception-ResNetV2 architecture was used and fully trained using the training set or partly retrained using the new-training set with clinical data; (**B**, iv), classifications were performed on images from an independent validation set, and the values were finally aggregated per patient to extract the T-SNE and the statistics; (**B**, v), Clinical data was encoded using one-hot encoding as preparation for three-way malignancy classifier. HH, Hepatic hemangioma; Nodules, other benign nodules; Metastatic, Metastatic malignancy from other sites; Primary, Primary malignancy except HCC.

Our CNN computational strategy is demonstrated in Figure 1. In the training set, liver tumors were marked in T2 sequences by trained senior abdominal radiologists based on formal diagnostic reports. Then, six images from six sequences (T2, diffusion, Pre-contrast T1, late arterial, portal venous, and equilibrium phase) were obtained for each cross section of the lesion by processing the images. The medical history and laboratory test results of individuals with malignant tumors were searched from the medical record system and encoded by the auto encoder to obtain clinical data, including age, gender, cirrhosis-related history, other cancers, tumor marker(AFP, CEA,CA-125,CA19-9, PSA, Ferritin), and liver function (albumin, total bilirubin, prolonged prothrombin time, hepatic encephalopathy, ascites). The coding table is in Table S3. The images and clinical information can be used as direct input to the neural networks according to different tasks (Figure 1). Based on the computational strategy outlined in Figure 1, three group models were trained. First, CNNs were developed to classify images into seven categories. Model A and B used six sequences and three un enhanced sequences (T2, diffusion, Pre-contrast T1) to train CNN, respectively. Second, six sequences (Model C) and three unenhanced sequences (Model D) were utilized to train CNNs for binary classification of benign and malignant. Third, malignant cases with complete clinical data in the training set were selected as a new training set and the integral computational pipeline (Figure 1) was applied to train new models in order to classify malignant tumors in to three categories. Model F and Model G, respectively, utilized six and three sequences alongside clinical data as the direct input, while Model E utilized only six imaging sequences as input. The 5-fold cross-validation results during training were reported in Supplementary Materials-Data File S1. Then three experienced radiologists were asked to independently classify MRI images in the validation set through the Picture Archiving and Communication Systems (PACS). They could refer to other information about patients such as their medical history, laboratory tests, and so on. This design could better reflect the true level of doctors in daily clinical situation than previous works (16, 23–25) that asked doctors to make judgements only based on images. When the results of the three radiologists were inconsistent, then they discussed together in order to get a diagnoses referred to as reader consensus.

### Deep-Learning Models in Seven-Way Classification Diagnosis

**Figure 2:**
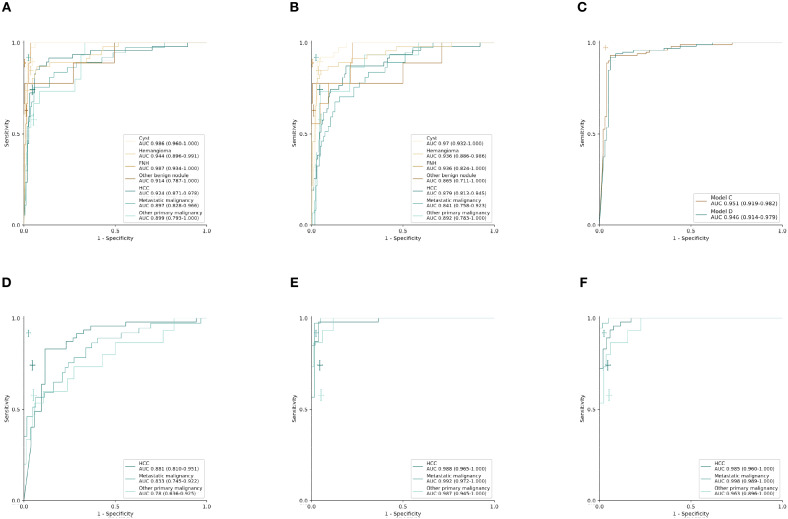
Performance of CNN models and radiologists in external validation set. **(A–C)** Receiver operating characteristic (ROC) curves in the validation set (*n* = 201 patients). **(A)** Model A: seven-way classifier with six sequences. **(B)** Model B: seven-way classifier with three unenhanced sequences. **(C)** Model C,D: binary classifier for benign and malignancy with six sequences and three unenhanced sequences. **(D–F)** ROC curves in the new validation set of malignant tumors (*n* = 99 patients). **(D–F)**, **(D)** Model E: three-way classifier with six sequences. **(E)** Model F: three-way classifier with six sequences and clinical data. **(F)** Model G: three-way classifier with three sequences and clinical data. The crosses indicate the performance of average radiologists for each category, the length of the cross represents the confidence Interval (CI).

Using the computational pipeline of Figure 1, Inception-ResNet V2 was first trained to classify liver tumors in to seven categories according to clinical practical criterion and tested in the validation cohort. Model A with six sequences achieved a high performance in seven-way classification in the validation set (Figure 2A), with AUC values for each category ranging from 0.897 (95% CI 0.828–0.966, metastatic malignancy) to 0.987 (95% CI 0.934–1.000, FNH), which was better than Model B with three unenhanced sequences (Figure 2B), although no significant difference existed (Table 1). Compared with the average level of readers, Model A achieved a competitive level for most categories, but for metastatic malignancy, the average reader seemed to perform better than Model A (Figure 2A). The performance between the model and reader consensus was further compared (Table 1). The model sensitivity for seven categories ranged from 53.3% (95% CI 26.6–78.7%, other primary malignancy) to 100% (95% CI 66.4–100%, FNH). The sensitivity of reader consensus for seven categories ranged from 55.6% (95% CI 21.2–86.3%, benign nodules) to 94.7% (95% CI 82.3–99.4%, FNH). There was no significant difference (*p* > 0.05) in the sensitivity for each category between Model A and reader consensus, except for metastatic malignancy (*P* = 0.003). In this category, reader consensus had a better performance than Model A because doctors could refer to clinical information including related medical history and laboratory test results, which are of great value for the diagnosis of metastatic tumors. For seven categories, the specificity of model A and reader consensus ranged from 91.6% (95% CI 86.0–95.4%, HCC) to 99.5% (95% CI 97.1–100%, benign nodules) and 94.8% (95% CI 90.0–97.7%, HCC) to 100% (95% CI 98.1–100%, FNH), respectively. Among all categories, only the specificity of FNH demonstrated a significant difference (*P* = 0.008) between Model A and reader consensus. Model A showed a higher sensitivity of 77.8% (95% CI 40.0–97.2%) and specificity of 99.5% (95% CI 97.1–100%) than reader consensus for benign nodules. For benign nodules, the number of patients with an accurate diagnosis by models was more than every experienced radiologist (Figure S2 for confusion matrix). Seven cases of cirrhosis nodules were all predicted correctly, indicating that the network performs well in differentiating high-risk cirrhosis nodules from HCC. Two wrongly predicted cases of benign nodules were confirmed as bile duct adenoma (predicted HCC) and angiomyolipoma (predicted HCC), respectively (Figure S3). HCC false negative cases possessed some common features, such as small tumor sizes or being highly differentiated (Figure S4). Those wrongly predicted cases were associated with a lack of enough similar cases in the training set, which may mean the network was not fully trained.

**Table 1:** Diagnostic performance of seven-way classifiers and radiologists in the validation set.

	**Value (95%CI)**			**McNemar's *P-*value^**a**^**	**Delong *P-*value**
	**Model A**	**Model B**	**Reader consensus**	**Model A vs. Reader**	**Model A vs. Model B**
**Cyst**					
AUC	0.986 (0.960, 1.000)	0.970 (0.932, 1.000)			0.147
Sensitivity, %	89.5 (75.2, 97.1)	89.5 (75.2, 97.1)	94.7 (82.3, 99.4)	0.688	
Specificity, %	96.9 (93.0, 99.0)	96.9 (93.0, 99.0)	98.2 (94.7, 99.6)	0.727	
**Hemangioma**					
AUC	0.944 (0.897, 0.991)	0.936 (0.886, 0.986)			0.66
Sensitivity, %	82.6 (68.6, 92.2)	87.0 (73.7, 95.1)	89.1 (76.4, 96.4)	0.508	
Specificity, %	95.5 (90.9, 98.2)	93.6 (88.5, 96.9)	98.1 (94.5, 99.6)	0.344	
**FNH**					
AUC	0.987 (0.934, 1.000)	0.936 (0.824, 1)			0.102
Sensitivity, %	100 (66.4, 100)	66.7 (29.9, 92.5)	88.9 (51.8, 99.7)	1.000	
Specificity, %	95.8 (92.0, 98.2)	94.8 (90.63, 97.5)	100 (98.1, 100)	0.008	
**Benign nodules**					
AUC	0.914 (0.787, 1.000)	0.865 (0.711, 1.000)			0.139
Sensitivity, %	77.8 (40.0, 97.2)	66.7 (29.9, 92.5)	55.6 (21.2, 86.3)	0.500	
Specificity, %	99.5 (97.1, 100)	99.5 (97.1, 100)	98.4 (95.5, 99.7)	0.625	
**HCC**					
AUC	0.925 (0.871, 0.978)	0.879 (0.813, 0.9452)			0.137
Sensitivity, %	87.2 (74.3, 95.2)	74.5 (59.7, 86.1)	87.2 (74.3, 95.2)	1.000	
Specificity, %	91.6 (86.0, 95.4)	86.4 (79.9, 91.4)	94.8 (90.0, 97.7)	0.267	
**Metastatic malignancy**					
AUC	0.897 (0.828, 0.966)	0.841 (0.758, 0.923)			0.039
Sensitivity, %	59.6 (42.1, 75.3)	40.5 (24.8, 57.9)	89.2 (74.6, 97.0)	0.003	
Specificity, %	97.6 (93.9, 99.3)	97.0 (93.0, 99.0)	97.6 (93.9, 99.3)	1.000	
**Primary malignancy except HCC**					
AUC	0.899 (0.793, 1.000)	0.892 (0.783, 1.000)			0.844
Sensitivity, %	53.3 (26.6, 78.7)	46.7 (21.3, 73.4)	60.0 (32.3, 83.7)	0.688	
Specificity, %	97.9 (94.6, 99.4)	96.8 (93.1, 98.8)	96.2 (92.4, 98.5)	0.727	

a *P-value was calculated between Model A (seven-classification) vs. Reader consensus using the McNemar's test*.

**Figure 3:**
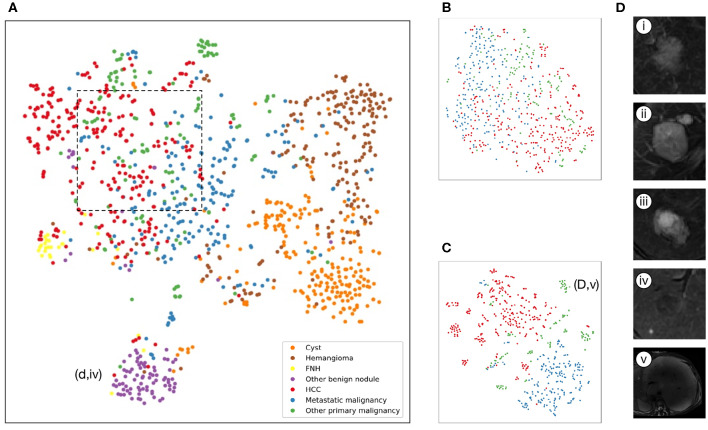
Illustration of classifiers learned by deep-learning projected to 2 dimensions for visualization via the t-SNE algorithm using values of the last fully connected layer in the CNNs of the validation set. **(A–C)**, Scatterplots where each point represents an image of lesions and the color represents the true category, show how the algorithm clusters. **(A)**, Model A: seven-way classifier with six sequences images, shows that seven clusters of the same clinical classes, and we can see benign tumor clusters are better than that of three malignant tumors. The purple point clouds(benign nodules) are effectively divided from red point clouds (HCC). **(B)**, Model E: three-way classifier with six sequences images for malignant tumors. **(C)**, Model G: three-way classifier with three sequences images and clinical data for malignant tumors, shows that three different color point clouds are more effectively clustered than Model E. **(D)** Insets of T2 images show some categories. (i), Hepatocellular carcinoma (ii), Metastatic malignant tumors from pancreas (iii), Intrahepatic cholangiocarcinoma (iv) DN that are difficult to identify with HCC. (v) malignant fibrous histiocytoma represented by outlier point clouds of c(d,v).

**Figure 4:**
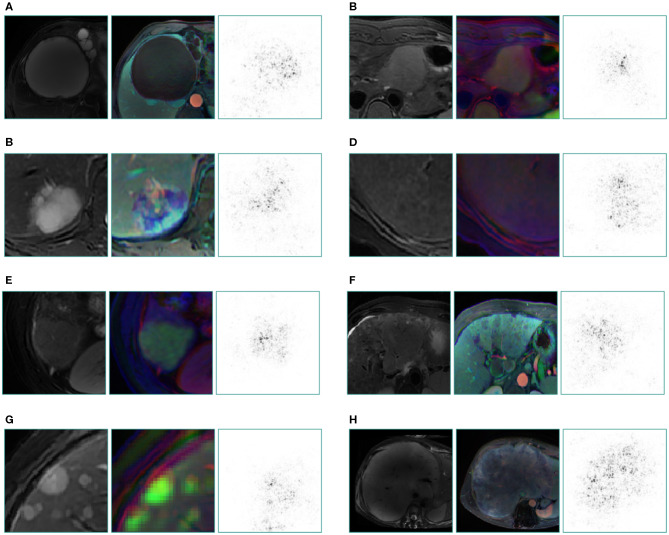
Saliency map for example images from seven categories of the validation set and a special case which not appeared in training set. These maps reveal the pixels that most influence a CNN's prediction. Saliency maps show the pixel gradients with relative to the CNN loss function. Darker pixels represent pixels with greater influence. Clear correlation between lesions and saliency maps are revealed. We selected T2 image as the original control, the middle is reconstructed image of three sequences (the left column is from three plain scan sequences, the right column is from three enhanced sequences), the right is a corresponding saliency map. **(A)** cyst, **(B)** FNH, **(C)** hemangioma, **(D)** benign nodule, **(E)** HCC, **(F)** primary adenocarcinoma, **(G)** metastatic malignancy originating from pancreas, **(H)** malignant fibrous histiocytoma, which still gains a good display although this rare type did not appear in the training set.

The internal features learned by the CNN using t-SNE (t-distributed Stochastic Neighbor Embedding) (36) were examined (Figure 3). Each point represents a group of liver tumor images from six different sequences projected from the 2048-dimensional output of the CNN's last hidden layer into two dimensions. For benign tumors, we observe clusters of points of the same clinical category clustered together (Figure 3A), whereas the point distributions of three categories of malignant tumors were not very clearly separated, which is consistent with the observation in the statistical indicators. Figure 4 shows saliency maps that identify the pixels on which the Inception-ResNet V2 model was fixating its attention on for prediction. As is seen, the network fixates most of its attention on the liver lesions themselves and ignores the background, which is in line with the clinical implication that the lesion and nearby region are more informative of diagnosis. However, the patterns are not specific enough to extract traditional radiologic features, and overall, the saliency map suggests that the deep learning model considered the most important regions when making the prediction, as presented in Figure 4.

### Deep-Learning Models in Malignancy Diagnosis

Model D was trained on the more challenging task of distinguishing benign and malignant tumors using only using three unenhanced sequences, which exhibited comparable performance to Model C with six sequences. Validated in the independent set, the AUC value was 0.946 (95% CI 0.914–0.979) for Model D and 0.951(95% CI 0.919–0.982) for Model C, but two ROC curves exhibited no significant difference in Delong's test (*P* = 0.664), which demonstrated that Model C and Model D have similar performances (Figure 2C). The sensitivity of Model C and Model D was 91.9% (95% CI 84.7–96.5%) and 90.9% (95% CI 83.4–95.8%), respectively, which were slightly lower than 99.0% (95% CI 94.5–100%) of reader consensus, but did not reach statistical significance (*P* = 0.375 and 0.219, respectively, estimated by the McNemar's test using binomial distribution). Specificity of Model C and Model D also had no significant difference compared with consensus (*P* = 0.549 and 0.754, respectively) (Table 2). In Model D, 93.6% (44/47) of HCC was correctly predicted as a malignancy, while the whole seven individuals of RN/DN nodules were all correctly predicted as benign. It showed that the network without enhanced images can effectively differentiate malignant from benign tumors, even for HCC and high-risk cirrhosis nodules that are difficult to distinguish in traditional HCC diagnostic imaging frameworks, such as LI-RADS, in the absence of contrast agents. As for atypical HCC that were wrongly predicted (very small lesions, benign tumors with carcinogenesis, highly differentiated, etc.), more cases for training are needed.

**Table 2:** Diagnostic performance of binary classifiers and radiologists in the validation set.

	**Value (95%CI)**			**McNemar's** ***P-*****value**		**Delong *P-*value**
	**Model C**	**Model D**	**Reader consensus**	**Model C vs. Reader**	**Model D vs. Reader**	**Model C vs. Model D**
AUC	0.951 (0.919, 0.982)	0.9416 (0.914, 0.979)				0.664
Sensitivity, %	91.9 (84.7, 96.5)	90.9 (83.4, 95.8)	89.1 (76.4, 96.4)	0.375	0.219	
Specificity, %	94.1 (87.6, 97.8)	94.1 (87.6, 97.8)	90.4 (79.0, 96.8)	0.549	0.754	

### Deep-Learning Models in Three-Way Classification Diagnosis for Malignancy

An approach that joined clinical data to CNN resulted in a much higher performance in Model F and Model G (Figures 2E,F, Table 3). The AUCs of Model G improved to 0.985 (95% CI 0.960–1.000, HCC), 0.998 (95% CI 0.989–1.000, metastatic malignancy), and 0.963 (95% CI 0.896–1.000, other primary malignancy), which were significantly better than those of 0.881 (95% CI 0.810–0.951), 0.833 (95% CI 0.745–0.922) and 0.780 (95% CI 0.636–0.925) in Model E (Figure 2D) with six sequences (*P*-values of 0.002, 0.0002, and 0.008, respectively). However, no statistical significance was observed in each category between Model F and Model G (*P*-values of 0.002, 0.0002, and 0.008, respectively), which demonstrated that after adding clinical data, classifiers with six sequences and three sequences had similar performances. Sensitivity and specificity of each category in Model G had no statistical significance compared with those of reader consensus (Table 3). Among three categories, the latter two included many specific tumors from different sites and histopathological sources (Table S2). The CNN network with the new approach is highly inclusive with the tumor type complexity. From t-SNE visualization of the last hidden layer representations of Model G and Model E (Figures 3B,C), clusters of points which belonged to the same clinical category were better gathered together in Model G than Model E, therefore, this also demonstrated that the modified end-to-end CNN with unenhanced images and clinical data as collaborative inputs achieves better classification performances than the network using enhanced images.

**Table 3:** Diagnostic performance of three-way classifiers and radiologists in the malignancy validation set.

	**Value (95%CI)**				**McNemar's *P-*Value^**a**^**	**Delong** ***P-*****Value**	
	**Model E**	**Model F**	**Model G**	**Reader consensus**	**Model G vs. Reader**	**Model E vs. Model G**	**Model F vs. Model G**
**HCC**							
AUC	0.879 (0.808, 0.949)	0.972 (0.938, 1.000)	0.951 (0.906, 0.997)			0.002	0.792
Sensitivity, %	93.6 (82.5, 98.7)	95.7 (85.5, 99.5)	95.7 (85.5, 99.5)	89.1 (76.4, 96.4)	0.289		
Specificity, %	67.3 (52.9, 79.7)	96.2 (86.8, 99.5)	90.4 (79.0, 96.8)	90.4 (79.0, 96.8)	0.063		
**Metastatic malignancy**							
AUC	0.814 (0.722, 0.907)	0.980 (0.947, 1.000)	0.985 (0.958, 1.000)			0.0002	0.403
Sensitivity, %	59.5 (42.1, 75.3)	100 (90.5, 100)	94.6 (81.8, 99.3)	89.2 (74.6, 97.0)	0.688		
Specificity, %	93.6 (84.3, 98.2)	96.8 (88.8, 99.6)	100 (94.2, 100)	95.1 (86.3, 99.0)	1.000		
**Primary malignancy except HCC**							
AUC	0.761 (0.613, 0.909)	0.989 (0.951, 1.000)	0.905 (0.801, 1.000)			0.008	0.081
Sensitivity, %	53.3 (26.6, 78.7)	86.7 (59.5, 98.3)	73.3 (44.,9 92.2)	60.0 (32.3, 83.7)	0.688		
Specificity, %	95.2 (88.3, 98.7)	100 (95.7, 100)	96.4 (89.9, 99.3)	91.6 (83.4, 96.5)	0.250		

a *P value was calculated between Model G (three-sequence images + clinical data) vs. Reader consensus using the McNemar's test*.

### Consistency Evaluation Between Models/Radiologists and Gold Standard

The agreement was then measured comparing deep-learning models, and the radiologists' consensus with the pathological/formal reports using Cohen's Kappa statistic (Table 4). It was observed that the agreement of all deep-learning models and radiologists with pathological/formal reports reached statistical significance (*P* < 0.01, estimated by the two-sample two-tailed z-test score), indicating the consistency between them. According to the interpretation Cohen suggested about the Kappa results (34), Model A had substantial consistency (kappa > 0.6), while Model C, Model D, Model E, and Model F had almost perfect consistency compared with the gold standard(kappa > 0.8). Regarding time spent, it can take a radiologist several minutes to analyze a patient's imaging depending on the difficulty of each individual, but deep-learning models just need a few seconds.

**Table 4:** Consistency analysis of models and radiologists compared with pathological or formal report.

	**Accuracy**	**Kappa**	**Z score**	***P-*Value**
Model A	79.1 (72.8, 84.5)	0.744	22.9	<0.01
Model B	71.1 (64.4, 77.3)	0.649	20.0	<0.01
Model C	93.5 (89,2, 96.5)	0.861	12.2	<0.01
Model D	93.0 (88.6, 96.1)	0.851	12.1	<0.01
Model E	72.7 (62.9, 81.2)	0.541	7.31	<0.01
Model F	93.9 (87.3, 97.7)	0.901	11.8	<0.01
Model G	91.9 (84.7, 96.5)	0.867	11.4	<0.01
Reader 1	88.1 (82.8, 92.2)	0.853	25.7	<0.01
Reader 2	77.1 (70.7, 82.7)	0.723	22.8	<0.01
Reader 3	84.6 (78.8, 89.3)	0.810	24.4	<0.01
Reader consensus	86.1 (80.5, 90.5)	0.829	25.1	<0.01

## Discussion

The findings of the current study show the feasibility and potential superiority of the integrated DLS in liver tumor MRI diagnosis in clinical situations. Deep learning achieves a performance on par with experienced radiologists in classifying liver tumors to seven categories. Utilizing unenhanced sequences, DLS can distinguish malignant from benign tumors, and then by combining medical texts and laboratory test results lead to a precise diagnosis for malignant tumors, even better than three experienced radiologists who have considered enhanced sequences. To the best of our knowledge, this is the largest study in the field of deep-learning-guided liver tumor diagnosis based on MR images worldwide, which has the most variable types of focal liver lesions (only inflammatory lesions excluded).

Evaluations of MR images by radiologists are generally subjective and are possibly influenced by their experience to an extent (40), even in LI-RADS which is majorly applicable to patients at high risk for HCC. Deep learning models have advantages in overcoming these problems. CNNs learning feature representations with an automated procedure and the interpretation maintains consistency and, therefore, diagnostic reproducibility. Thus, for developing countries such as China or other undeveloped countries, where there is an unbalanced distribution of medical resources between urban and rural areas, the deep-learning models could help in bridging the diagnosis gap of MRI between national hospitals and primary care hospitals, which can also be served as a quick and reliable opinion for junior radiologists in the diagnosis of hepatic lesions.

The confusion matrix (Figure S2) shows that both the seven-way classifier and doctors have poor performances in malignant classification, especially with HCC and other primary malignancies that are difficult to distinguish from each other, which may be because that intrahepatic cholangiocarcinoma have some similar imaging manifestations with HCC. It is worth noting that some rare types, such as malignant fibrous histiocytoma which belongs to other primary malignancies, have not been fully studied in the training set due to a lack of sufficient cases. The same situation exists with metastatic tumors with different origins such as pancreatic neuroendocrine cancer, lung squamous cell carcinoma, and so on. The inclusion of these individuals to the study reduced the diagnosis efficacy of the model, however, considering the clinical application scenarios, we did not exclude these individuals. After adding clinical data, the performance of three-way CNNs are greatly accelerated with an accuracy over 90%, which is better than radiologists, even in the model utilizing unenhanced sequences. Moreover, the binary CNN with unenhanced images exhibits almost the same performance with that using enhanced sequences and the prediction accuracy for seven RN/DN and HCC achieved 100% and over 90%, respectively. These results indicated that deep learning can mine more information in unenhanced sequences and clinical data to make judgments than human experts. Therefore, CNN has the potential ability to use non-contrast MRI to make diagnoses of liver tumors, even for high-risk cirrhotic nodules. This advantage could protect patients from potential gadolinium unsafety, especially for those allergic to contrast agents or those who cannot tolerate it with liver or kidney failure. Considering the high expenditure of gadolinium and hepatobiliary contrast agents, non-contrast MRI could be served as a potential cost-effective screening and surveillance tool (12) for high-risk patients under the assistance of DLS. More patients with cirrhotic nodules and small HCCs will be included in future multi-center prospective collaborative research and the DLS will be further validated.

In addition, two false negative cases in puncture biopsies were selected to be validated separately, which were not included in the training or test set. Combined with the patient's medical history and blood test results, they were still considered as malignant and were verified in subsequent treatment (Figure S5). But the seven-way CNNs and binary CNNs suggested they were malignant. Image-based features represent the phenotype of the entire tumor in three dimensions and not just the portion that was punctured for pathological testing (11, 17), and thereby can assess the condition of the entire lesion and yield more comprehensive and accurate results (41), which is another advantage of CNN-based image recognition.

Many previous studies have gained impressive achievements in medical image classification using deep neural networks, such as in the classification of skin cancer (20), lung cancer screening (21), diabetic retinopathy examination (22), liver fibrosis assessment (42–44), etc. However, owing to the diversity and complexity of liver masses, there are only a few studies (16) that applied deep learning for multi-classification of liver tumors using CT (23) and MRI (16, 24, 25). Hamm et al. (16) selected six common specific hepatic lesion types (*n* = 494 patients) and utilized enhanced sequences to train the model. The model was not validated in an independent set. The study of Yamashita et al. (25) was also based on enhanced sequences in a small dataset. Whereas, our study evaluated the value of non-enhanced sequences (T2, diffusion, T1 pre-contrast) and achieved good performances in a binary model, which has the potential to reduce high costs and the risk of contrast-related side effects. Moreover, different from all previous studies, we modified the input layer to receive a variety of data input and used the concatenate operation to combine image features with clinical data features, and then classified liver tumors through a fully connected layer. This end-to-end deep learning model with clinical data and images can fully utilize comprehensive information to improve diagnostic performance.

The DLS is applicable for patients with all types of liver tumors, except inflammatory lesions. The MR images used in the study were gained from different MRI scanners and acquisition protocols, which contributed to increased data diversity and heterogeneousness in training the algorithm and demonstrated the robustness of models. Therefore, once the DLS is established, radiologists just need to perform a standardized selection of ROI for liver tumors on a T2 sequence in the daily workflow of MRI reading to conduct such analysis, which is extremely convenient and timesaving for clinical applications. We are working on constructing a cloud-based multi-institutional artificial intelligence platform and a user-friendly website to provide freely accessible telemedical assistance for clinicians to accelerate the interpretation of MR images, meanwhile, to collect the related follow-up pathological information and feed it back to DLS so as to continuously improve its performance.

Our study has several limitations, which should be acknowledged. First, the study is a single-center retrospective study, although the validation set has maximally simulated the scenario of a clinical practice, multicenter prospective research is still necessary to evaluate performance in a real-world, clinical setting. Second, more patients with some specific types of focal liver diseases (RN, DN, small HCC, HCC without pathology, inflammation, etc.) need to be included in future training, in order to be applicable across the full distribution and spectrum of lesions encountered in a clinical practice. Third is a potential problem for medical applications about the interpretability of “black box” algorithms (45). In the current study, darker pixel regions in the saliency map and clusters of point clouds in t-SNE revealed that CNN prediction at least follows some aspects of human expert knowledge, which can be seen as an application of interpretable deep-learning on multimodal medical data, in addition, research into explainable AI and evaluation of interpretability is occurring at a rapid pace (46, 47).

In the future, we would ideally include some types of less common liver masses, such as abscesses, adenomas, rare malignancies, etc. Importantly, further high-quality, prospective, multicenter studies will also be performed, especially for high-risk patients with cirrhosis. For these patients, the main differentials for small HCC are benign regenerative/ dysplastic nodules and pre-malignant nodules. These are quite challenging clinically. This is an inherent problem in retrospective liver nodule research as most of these will not be confirmed histologically, however many of these nodules when followed turn out to have been early malignancy. Therefore, early diagnosis of these premalignant nodules may be the most important value that deep learning networks can offer over human experts, which would have to be explored by a rigorously designed prospective study. On the other hand, we will explore the utilization of more ideal data visualization tools (46–48) to allow further degrees of visual understanding of how algorithms make decisions, through identifying relevant imaging features and showing where these features are found on an image, so as to make the “black box” more transparent.

## Conclusions

In summary, we have developed a deep learning-based system which can supply a reliable and timesaving assisted diagnosis in a clinical setting by classifying liver tumors on MRI to seven categories with high accuracy. Meanwhile, it can use non-enhanced MRI to distinguish malignant tumors from benign tumors, and, after adding clinical data, it can provide accurate classification and diagnosis for malignant tumors, which could avoid contrast-related side effects and reduce costs. The DLS was trained with data in a various acquisition condition, and this classification system covers most types of liver tumors, which is unprecedented. All of these suggested a good potential of DLS for clinical generalization. Further prospective multicenter studies in larger patient populations and high-risk cirrhosis patients are still needed.

## Data Availability Statement

The data that support the findings of this study are available from the corresponding author upon reasonable request.

## Ethics Statement

The studies involving human participants were reviewed and approved by Independent institutional review boards of Sir Run Run Shaw Hospital. Written informed consent for participation was not required for this study in accordance with the national legislation and the institutional requirements.

## Author Contributions

SZ, WL, ZJ, JS, and YW created the datasets, interpreted the data, outlined ROI, and defined the clinical labels. MC, YJ, YY, and JQ developed the network architecture and data/modeling infrastructure, training, and testing setup. SZ and MC created the figures, wrote the methods, and performed additional analysis requested in the review process, and performed statistical analysis. SZ wrote the manuscript. XC, JL, and ZW provided clinical expertise and guidance on the study design. HL and YT advised on the modeling techniques. XC and HL supervised the project.

## Conflict of Interest

The authors declare that the research was conducted in the absence of any commercial or financial relationships that could be construed as a potential conflict of interest.

## Supplementary Material

Figure S1 Flowchart of the procedures in the selection of training and validation set.^*^Lesion-related treatment included surgery, transcatheter arterial chemoembolization, radiofrequency ablation, chemotherapy, radiotherapy, targeted drug therapy, etc. Number of patients with typical malignant imaging performance and in advanced stages without surgical indications, usually received therapy directly without biopsy. Reasons for exclusion included a unified method for preprocessing resulted in some images not being of a good enough quality owing to different parameters in multiple sequences of multiple machines from different manufacturers. For patients in the follow-up, 82 individuals were reviewed by ultrasound or radiology inspection in the following 6–10 months from the current examination date, and the diagnosis remains consistency.

Figure S2 Confusion matrix comparison between CNNs and radiologists. These show the number of patients for each class of gold standard and predicted class. The number of patients who were correctly predicted are found on the diagonal. **(A)** Confusion matrices for Model A and radiologists for the seven-way classification task in the validation set reveal similarities in misclassification between human experts and the CNN; confusion matrices for Model B, which performance is inferior to Model A. **(B)** Confusion matrices for Model E,F,G and radiologists for the three-way classification task in new validation set reveal the CNN with clinical data achieve higher performance than experienced experts.

Figure S3 Example HCC false positive cases. (i), Epithelioid angiomyolipoma. (ii), Bile duct adenoma. **(A)** T2WI **(B)** DWI **(C)** Precontrast-T1WI **(D)** late arterial phase **(E)** portal venous phase **(F)** equilibrium phase.

Figure S4 Example HCC false negative cases. (i). Hepatocellular adenoma with carcinogenesis. (ii), small hepatocellular carcinoma. **(A)** T2WI **(B)** DWI **(C)** Precontrast-T1WI **(D)** late arterial phase **(E)** portal venous phase **(F)** equilibrium phase.

Figure S5 Example false negative case in puncture biopsy report, actually positive in clinical assessment and verified in subsequent treatment. **(A–E)** T2-Weighted image of a lesion in the same section **(A)** Jan 14, 2017, histopathology report after biopsy showed: Nodular cirrhosis. But combined with history and tumor indicators, it was still considered that the risk of malignancy was extremely high and TACE was given. **(B,C)**,The second and third TACE were given in Feb 23, 2017 and May 18, 2017, the lesion shrunk enough to almost disappear. **(D,E)**, The lesion enlarged by the follow-up on Nov 21,2017 and Jan 18, 2018, which suggested it was a malignant tumor. **(F,G)** T2-Weighted image of the lesion of another case. Histopathology report after biopsy showed: Fibrous tissue hyperplasia, a small amount of shed atypical cells were seen. MRI report showed: Liver cancer with intrahepatic metastasis, left and right intrahepatic portal vein tumor thrombus. With comprehensive consideration, this case is a malignant tumor, but the local biopsy leads to a negative result because of partial necrosis of the lesion.

Table S1 Baseline characteristics of the training and validation set.

Table S2 Disease distribution of each category in the training and validation cohort.

Table S3 Medical text and laboratory data coding table.

Table S4 Imaging parameters in various sequences of magnetic resonance imaging.
