# Supplementary material for: Deep Learning for Accurate Diagnosis of Liver Tumor Based on Magnetic Resonance Imaging and Clinical Data
Source: Front Oncol. 2020 May 28;10:680. doi: 10.3389/fonc.2020.00680 (PMC7271965; doi:10.3389/fonc.2020.00680)
Supplement: Figure S1 — Flowchart of the procedures in the selection of training and validation set.*Lesion-related treatment included surgery, transcatheter arterial chemoembolization, radiofrequency ablation, chemotherapy, radiotherapy, targeted drug therapy, etc. Number of patients with typical malignant imaging performance and in advanced stages without surgical indications, usually received therapy directly without biopsy. Reasons for exclusion included a unified method for preprocessing resulted in some images not being of a good enough quality owing to different parameters in multiple sequences of multiple machines from different manufacturers. For patients in the follow-up, 82 individuals were reviewed by ultrasound or radiology inspection in the following 6–10 months from the current examination date, and the diagnosis remains consistency. [file Data_Sheet_2.pdf]

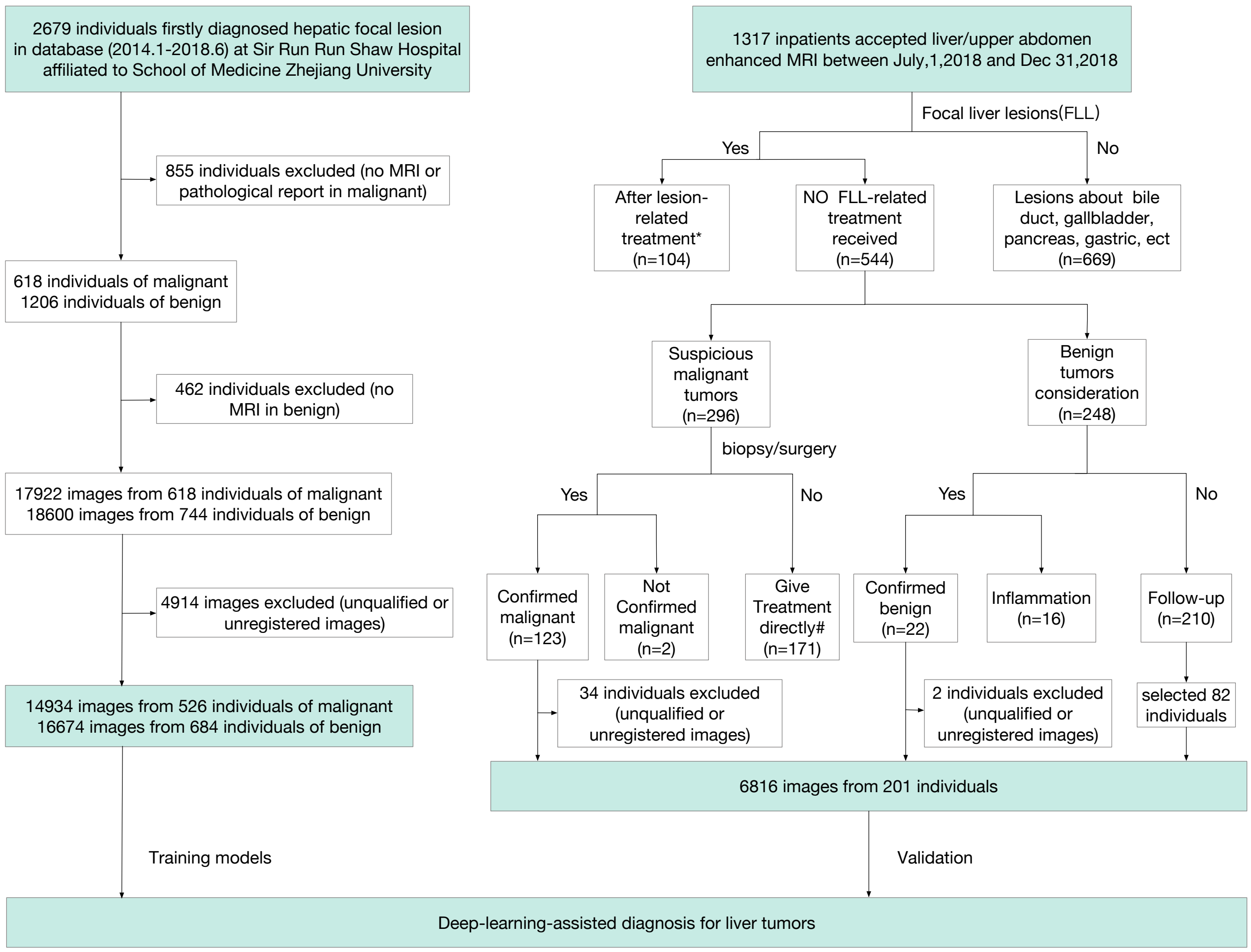

Fig. S1. Figure S1 | Flowchart of the procedures in the selection of training and validation set. ∗Lesion-related treatment included surgery, transcatheter arterial chemoembolization, radiofrequency ablation, chemotherapy, radiotherapy, targeted drug therapy, etc. Number of patients with typical malignant imaging performance and in advanced stages without surgical indications, usually received therapy directly without biopsy. Reasons for exclusion included a unified method for preprocessing resulted in some images not being of a good enough quality owing to different parameters in multiple sequences of multiple machines from different manufacturers. For patients in the follow-up, 82 individuals were reviewed by ultrasound or radiology inspection in the following 6–10 months from the current examination date, and the diagnosis remains consistency.

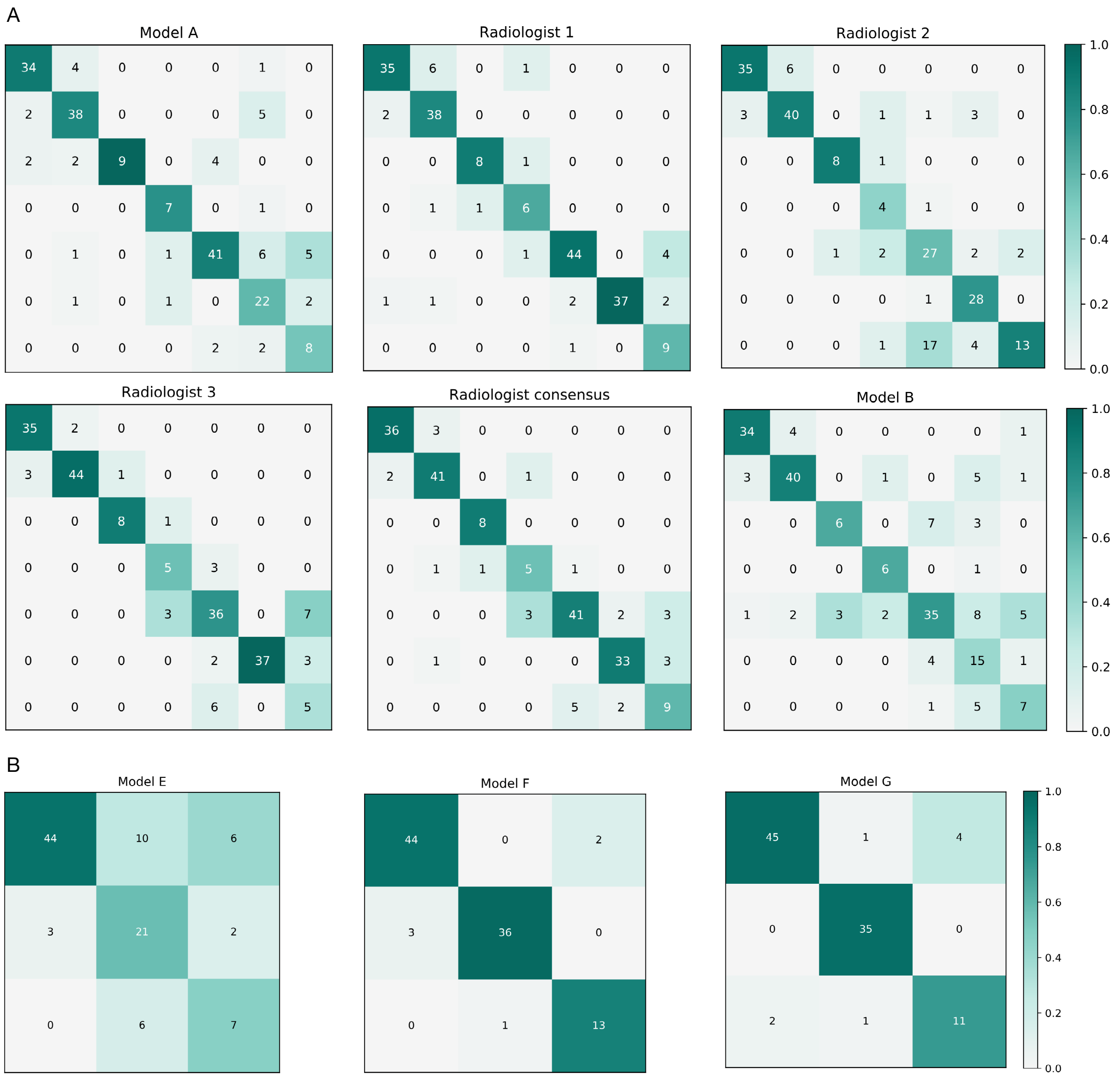

Fig. S2. Confusion matrix comparison between CNNs and radiologists. These show the number of patients for each class of gold standard and predicted class. The number of patients who were correctly predicted are found on the diagonal.(A) Confusion matrices for Model A and radiologists for the seven-way classification task in the validation set reveal similarities in misclassification between human experts and the CNN; confusion matrices for Model B, which performance is inferior to Model A. (B) Confusion matrices for Model E,F,G and radiologists for the three-way classification task in new validation set reveal the CNN with clinical data achieve higher performance than experienced experts.

i) Epithelioid angiomyolipoma

A. T2WI

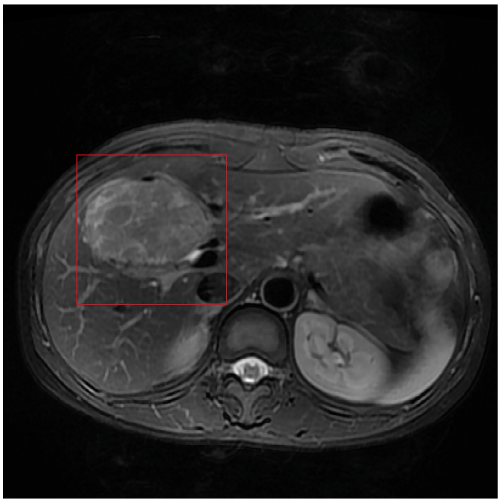

B. DWI

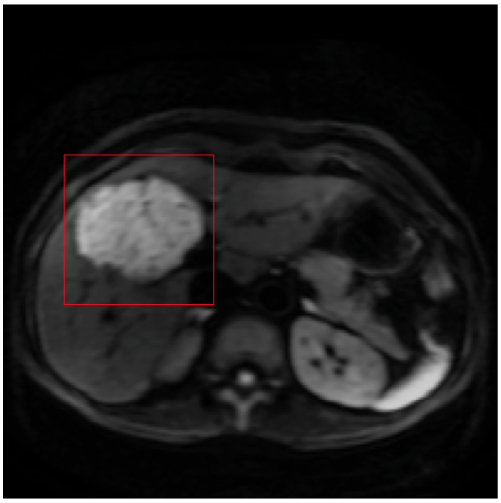

C. Precontrast—T1WI

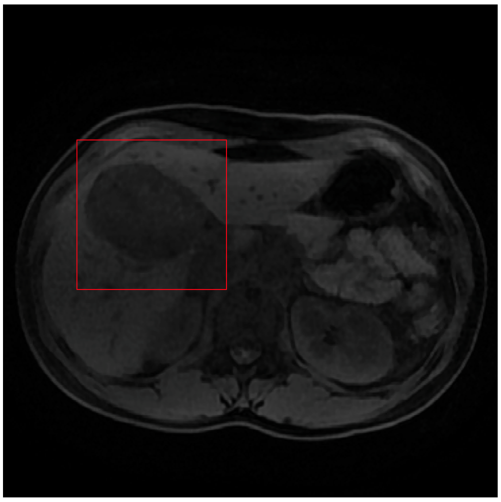

D. late arterial phase

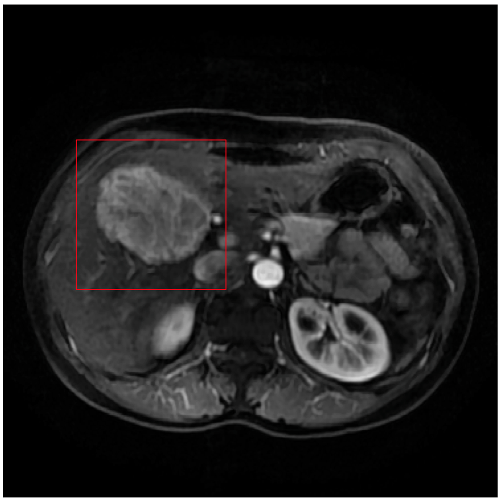

E. portal venous phase

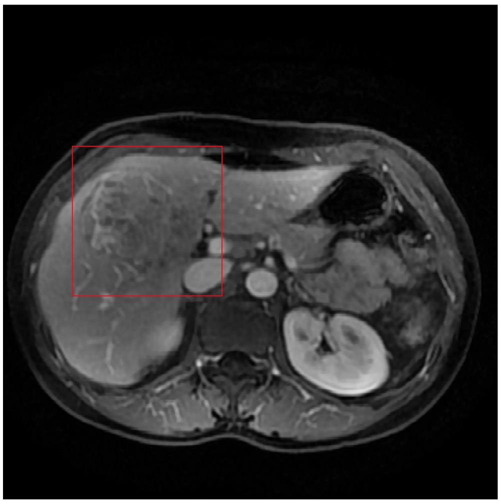

F. equilibrium phase

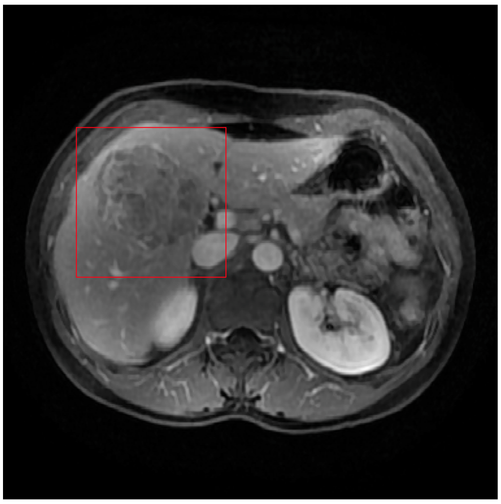

ii) Bile duct adenoma

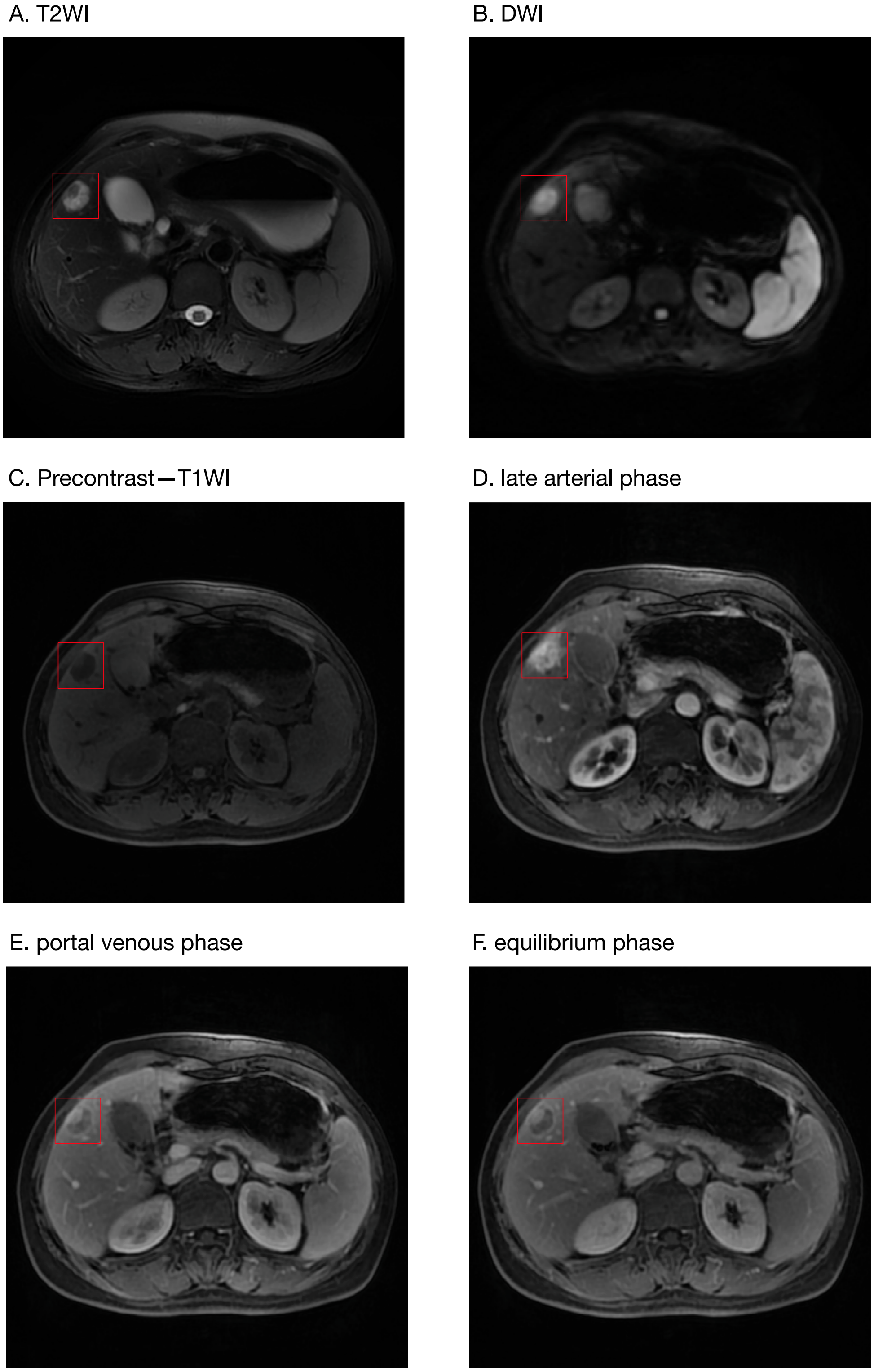

Fig. S3. Example HCC false positive cases. i), Epithelioid angiomyolipoma. ii), Bile duct adenoma. (A) T2WI (B) DWI (C) Pre-contrast—T1WI (D) late arterial phase (E) portal venous phase (F) equilibrium phase.
