# Supplementary material for: Deep Learning for Accurate Diagnosis of Liver Tumor Based on Magnetic Resonance Imaging and Clinical Data
Source: Front Oncol. 2020 May 28;10:680. doi: 10.3389/fonc.2020.00680 (PMC7271965; doi:10.3389/fonc.2020.00680)
Supplement: Figure S4 — Example HCC false negative cases. (i). Hepatocellular adenoma with carcinogenesis. (ii), small hepatocellular carcinoma. (A) T2WI (B) DWI (C) Precontrast-T1WI (D) late arterial phase (E) portal venous phase (F) equilibrium phase. [file Data_Sheet_3.pdf]

i) Hepatocellular adenoma with carcinogenesis

A. T2WI

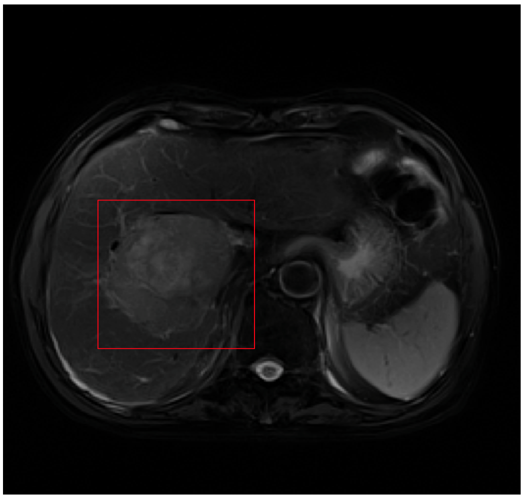

B. DWI

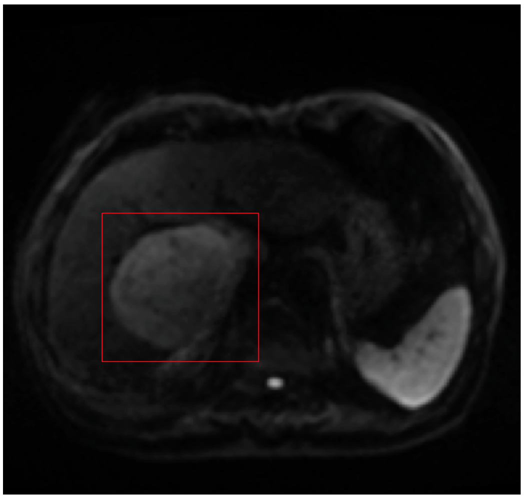

C. Precontrast—T1WI

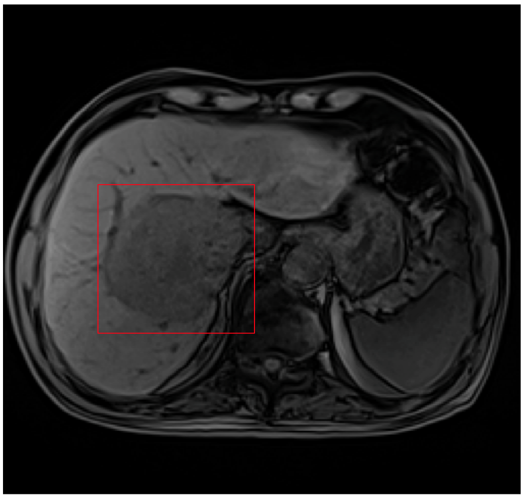

D. late arterial phase

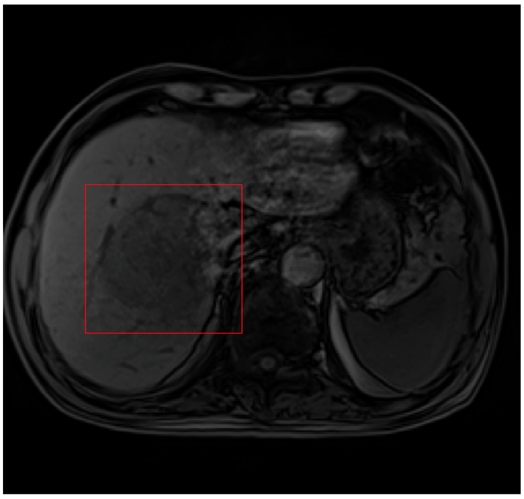

E. portal venous phase

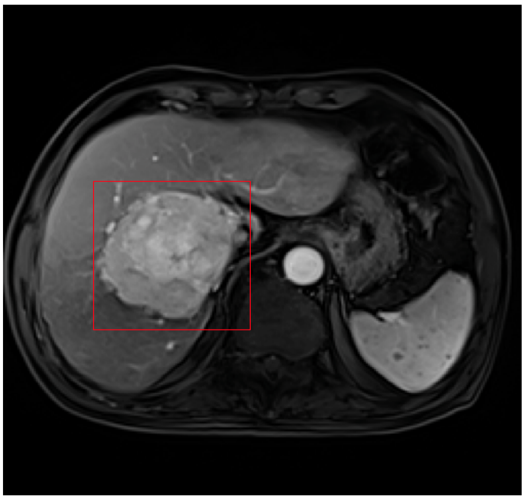

F. equilibrium phase

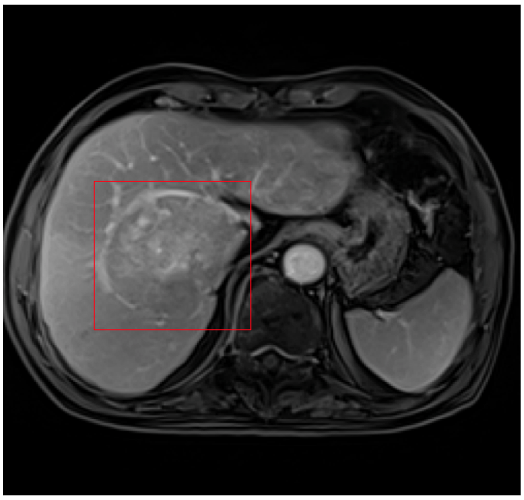

ii), small hepatocellular carcinoma

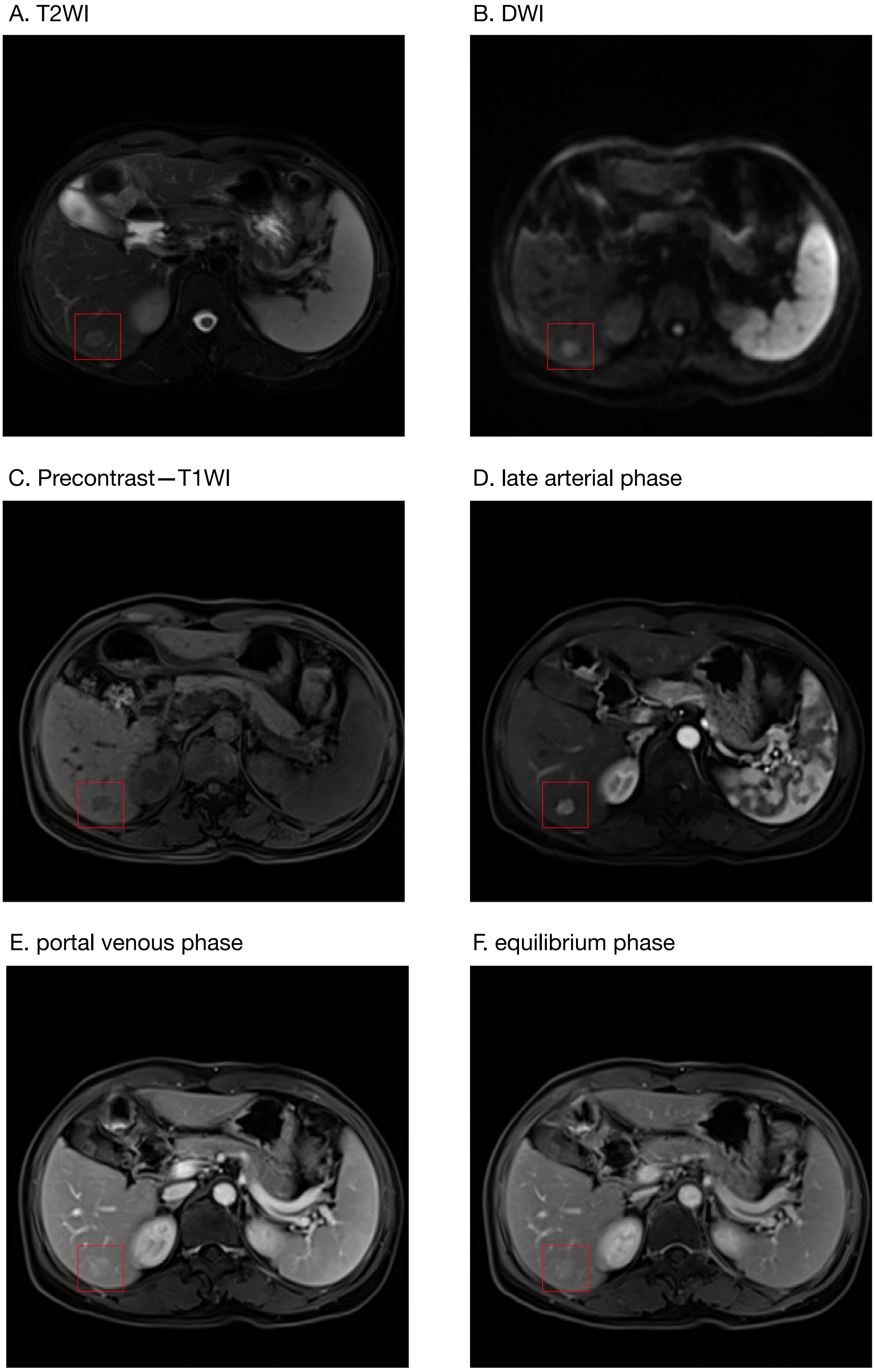

Fig. S4. Example HCC false negative cases. i). Hepatocellular adenoma with carcinogenesis. ii), small hepatocellular carcinoma. (A) T2WI (B) DWI (C) Pre-contrast—T1WI (D) late arterial phase (E) portal venous phase (F) equilibrium phase.

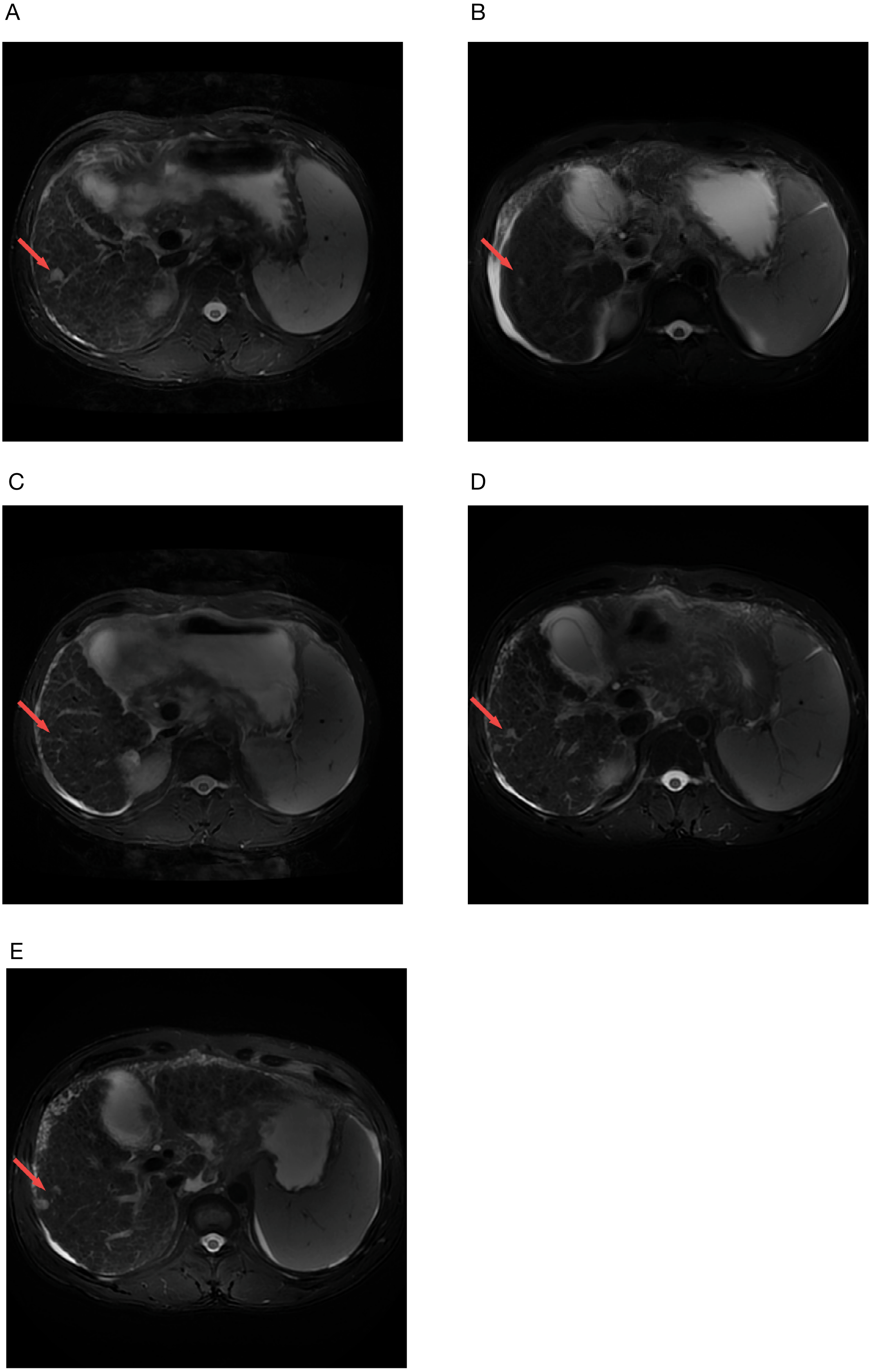
A
B
C
D
E

F G

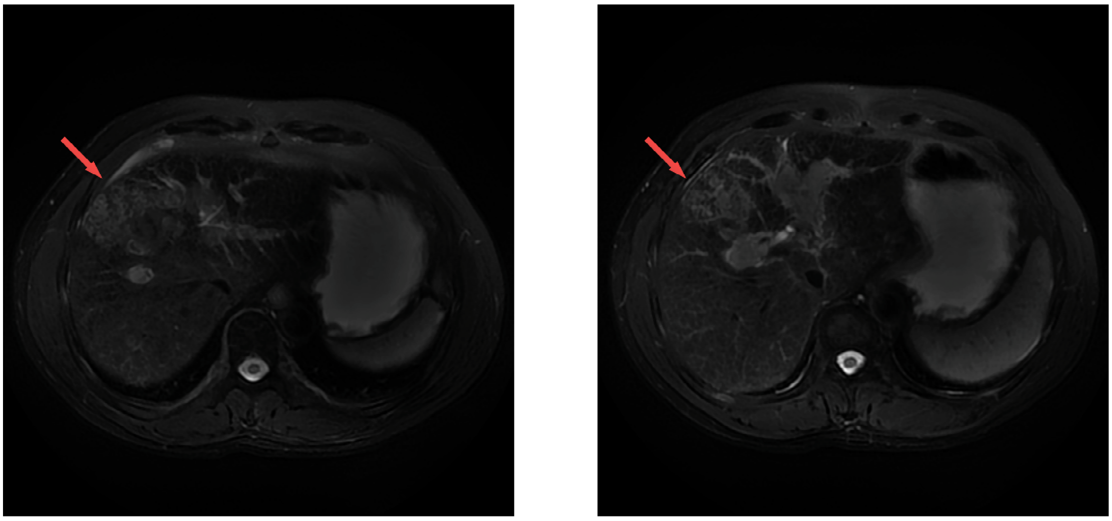

Fig. S5. Example false negative case in puncture biopsy report, actually positive in clinical assessment and verified in subsequent treatment. (A–E) T2-Weighted image of a lesion in the same section (A) Jan 14, 2017, histopathology report after biopsy showed: Nodular cirrhosis. But combined with history and tumor indicators, it was still considered that the risk of malignancy was extremely high and TACE was given. (B,C),The second and third TACE were given in Feb 23, 2017 and May 18, 2017, the lesion shrunk enough to almost disappear.(D,E), The lesion enlarged by the follow-up on Nov 21,2017 and Jan 18, 2018, which suggested it was a malignant tumor. (F,G) T2-Weighted image of the lesion of another case. Histopathology report after biopsy showed: Fibrous tissue hyperplasia, a small amount of shed atypical cells were seen. MRI report showed: Liver cancer with intrahepatic metastasis, left and right intrahepatic portal vein tumor thrombus. With comprehensive consideration, this case is a malignant tumor, but the local biopsy leads to a negative result because of partial necrosis of the lesion.
