# Supplementary material for: Deep Learning for Accurate Diagnosis of Liver Tumor Based on Magnetic Resonance Imaging and Clinical Data
Source: Front Oncol. 2020 May 28;10:680. doi: 10.3389/fonc.2020.00680 (PMC7271965; doi:10.3389/fonc.2020.00680)
Supplement: Table S1 — Baseline characteristics of the training and validation set. [file Data_Sheet_1.pdf]

Table S1 Baseline characteristics of the training and validation set

Variables	Cyst	Hemangiomas	FNH	Benign nodule	HCC	Metastatic malignancy	Other primary malignancy	Benign	Malignant	Total
Training set baseline characteristics										
Patients with pathology	17 (15%)	65 (28%)	41 (22%)	53 (36%)	337 (98%)	95 (100%)	87 (100%)	176 (26%)	519 (99%)	695 (57%)
images	1368	2796	918	864	8916	3156	2640	5946	14712	20658
Patients with image-diagnosis	100 (85%)	164 (72%)	149 (78%)	95 (64%)	7 (2%)	0 (0%)	0 (0%)	508 (74%)	7 (1%)	515 (43%)
images	2016	3900	2262	2550	222	0	0	10728	222	10950
Male	48 (41%)	85 (37%)	87 (46%)	114 (77%)	293 (85%)	48 (51%)	46 (53%)	334 (49%)	387 (74%)	721 (60%)
image	1272	2310	1482	2592	7698	1914	1398	7656	11010	18666
Female	69 (59%)	144 (63%)	103 (54%)	34 (23%)	51 (15%)	47 (49%)	41 (47%)	350 (51%)	139 (26%)	489 (40%)
images	2112	4386	1698	822	1440	1242	1242	9018	3924	12942
Age (years)	55 (26-84)	48 (20-85)	36 (17-73)	53 (20-80)	57 (19-88)	58 (30-82)	64 (29-82)	47 (17-85)	58 (19-88)	52 (17-88)
Age<=40 male	3 (3%)	22 (10%)	55 (29%)	18 (12%)	25 (7%)	6 (6%)	0 (0%)	98 (14%)	31 (6%)	129 (11%)
Age<=40 female	7 (6%)	36 (16%)	74 (39%)	2 (1%)	3 (1%)	2 (2%)	1 (1%)	119 (17%)	6 (1%)	125 (10%)
Age>40 male	45 (38%)	63 (28%)	32 (17%)	96 (65%)	268 (78%)	42 (44%)	46 (53%)	236 (35%)	356 (68%)	592 (49%)
Age>40 female	62 (53%)	108 (47%)	29 (15%)	32 (22%)	48 (14%)	45 (47%)	40 (46%)	231 (34%)	133 (25%)	364 (30%)
Data are n, n (%) or median (IQR). N= 1210 patients and 31608 images (5268 groups, 6 images from 6 different sequences per group)										
Validation set baseline characteristics										
Patients with pathology	1 (3%)	12 (26%)	5 (56%)	2 (22%)	47 (100%)	37 (100%)	15 (100%)	20 (20%)	99 (100%)	119 (59%)
images	78	642	108	54	1692	1224	654	882	3570	4452
Patients with image-diagnosis	37 (97%)	34 (74%)	4 (44%)	7 (78%)	0 (0%)	0 (0%)	0 (0%)	82 (80%)	0 (0%)	82 (41%)
images	1230	624	48	462	0	0	0	2364	0	2364
Male	26 (68%)	18 (39%)	5 (56%)	5 (56%)	38 (81%)	18 (49%)	9 (60%)	54 (53%)	65 (66%)	119 (59%)
image	870	330	60	414	1446	738	384	1674	2568	4242
Female	12 (32%)	28 (61%)	4 (44%)	4 (44%)	9 (19%)	19 (51%)	6 (40%)	48 (47%)	34 (34%)	82 (41%)
images	438	936	96	102	246	486	270	1572	1002	2574

Age (years)	62 (34-87)	53 (25-78)	32 (17-52)	55 (45-69)	59 (34-80)	58 (17-72)	58 (41-77)	55 (17-87)	58 (17-80)	56 (17-87)
Age<=40 male	1 (3%)	2 (4%)	3 (33%)	0 (0%)	2 (4%)	1 (3%)	0 (0%)	6 (6%)	3 (3%)	9 (4%)
Age<=40 female	1 (3%)	6 (13%)	4 (44%)	0 (0%)	1 (2%)	1 (3%)	0 (0%)	11 (11%)	2 (2%)	13 (6%)
Age>40 male	25 (66%)	16 (35%)	2 (22%)	5 (56%)	36 (77%)	17 (46%)	9 (60%)	48 (47%)	62 (63%)	110 (55%)
Age>40 female	11 (29%)	22 (48%)	0 (0%)	4 (44%)	8 (17%)	18 (49%)	6 (40%)	37 (36%)	32 (32%)	69 (34%)

Data are n, n (%) or median (IQR). N= 201 patients and 6816 images (1136 groups, 6 images from 6 different sequences per group)

Table S2 Disease distribution in each category

Disease distribution in each category of training set (n=1210 patients)	
cyst	simple hepatic cyst 115， cyst with bleeding 1， mucinous cystadenoma 1
Hemangioma	cavernous hemangioma 229
FNH	focal nodular hyperplasia of hepatocytes 190
Benign nodules	regenerative nodules 121, dysplasia nodules 26, epithelioid angiomyolipoma 1
HCC	hepatocellular carcinoma 344
Metastatic malignant tumors from other sites （primary cancer)	colorectal cancer 63， breast cancer 9， pancreatic cancer 5, pancreatic neuroendocrine carcinoma 2， lung cancer 3, gastric cancer 3, gallbladder cancer 3， cervical cancer 1， Uterine leiomyosarcoma 1， anal canal neuroendocrine carcinoma 1 , anal malignant melanoma 1 , skin melanoma 1 , right mandibular adenoid cystic carcinoma 1， retroperitoneal smooth sarcoma 1
Primary hepatic malignancies other than HCC	adenocarcinoma 54, intrahepatic cholangiocarcinoma 16, mixed hepatocellular carcinoma-cholangiocarcinoma 6， intrabiliary papilloma 5， sarcoma 3， neuroendocrine carcinoma 1， adenosquamous carcinoma 1， malignant perivascular epithelioid cell tumor（PEComa） 1
Disease distribution in each category of validation set (n=201 patients and cases indicated by black body did not appear in the training set.)	
cyst	simple hepatic cyst 37， multiple cystic lesions, complex cysts 1
Hemangioma	cavernous hemangioma 46
FNH	focal nodular hyperplasia of hepatocytes 9
Benign nodules	regenerative nodules 3 , dysplasia nodules 3, hamartoma 1, epithelioid angiomyolipoma 1, bile duct adenoma 1
HCC	47 hepatocellular carcinoma
Metastatic malignant tumors from other sites （primary cancer)	colorectal cancer 27， skin melanoma 2， pancreatic neuroendocrine carcinoma 2， breast cancer 2， lung cancer 3， ovarian cancer 1
Primary hepatic malignancies other than HCC	Primary hepatic malignancies other than HCC: adenocarcinoma 12, intrahepatic cholangiocarcinoma 1, neuroendocrine carcinoma 1， malignant fibrous histiocytoma 1

Table S3 Medical text and laboratory data coding table

Age(years)	<40 , [1,0]; >40,[0,1]
Gender	female , [1,0] ; male , [0,1]
Cirrhosis-related history	no , [1,0,0,0] ; HBV/HCV related , [0,1,0,0] ;<br>Alcoholic , [0,0,1,0] ; Obstructive , [0,0,0,1]
Other cancer	from liver and bile duct , [1,0] ; from other cites [0,1]
AFP (ng/mL)	<8.78 , [1,0,0] ; x<400 , [0,1,0] ; x>400 , [0,0,1]
CEA(ng/mL)	<5 , [1,0] ; >5 , [0,1]
CA-125(U/mL)	<35 , [1,0] ; >35 , [0,1]
CA19-9(IU/mL)	<37 , [1,0] ; >37 , [0,1]
PSA(ng/mL)	<4 , [1,0] ; >4 , [0,1]
Ferritin(μg/L)	<400 , [1,0]; >400 , [0,1]
Albumin(g/L)	<28 , [1,0,0] ; <35 , [0,1,0] ; > 35 , [0,0,1]
Total bilirubin(μmol/L)	<34 , [1,0,0] ; <51 , [0,1,0] ; > 51 , [0,0,1]
Prolonged prothrombin time(s)	<4 , [1,0,0] ; <6 , [0,1,0] ; > 6 , [0,0,1]
Hepatic encephalopathy(score)	<1 , [1,0,0] ; <3 , [0,1,0] ; > 4 , [0,0,1]
Ascites	Yes , [1,0] ; No , [0,1]

Table S4 Imaging parameters in various sequences of magnetic resonance imaging

Sequences	Imaging parameters
T1-weighted sequences	TR= 4–176 ms<br>TE= 1–3 ms<br>flip angle=10–70°<br>bandwidth= 62–1100 Hz<br>Slice thickness= 4–6 mm<br>Image matrix= 256 × 80 to 320 × 224 mm<br>field-of-view(FOV)=300 × 200 mm to 500 × 400 mm
T2-weighted sequences	TR=2000–6000 ms<br>TE=1-155 ms<br>flip angle=1–180°<br>bandwidth=40–800 Hz<br>slice thickness=6–8 mm<br>image matrix=256 × 100 to 320 × 256 mm<br>field-of-view(FOV)= 400 × 100 mm to 500 × 400 mm
Diffusion-weighted imaging<br>(b-values: 800 s/mm2)	TR= 4000-6000 ms<br>TE= 6–100 ms<br>flip angle=1–180°<br>bandwidth=60–2500 Hz<br>slice thickness=6–8 mm<br>image matrix =96 × 100 to 192× 130mm<br>field-of-view(FOV)= 300× 100 mm to 600× 400 mm

Data file S1:

The cross-validation results of three major models were as follows:

Model A: seven-way classifier using six sequences

Model D: binary classifier using three un-enhanced sequences（T2, DWI, T1-precontrast）

Model G: three-way classifier using three un-enhanced sequences（T2, DWI, T1-precontrast）and clinical data

The mean accuracy of five-fold cross-validation for models:

Model A:(0.7631+0.7621+0.7823+0.7751+0.7731)/5=0.7714

Model D: (0.8427+0.908+0.8790+0.9073+0.9034)/5=0.8881

Model G: (0.8447+0.8416+0.8519+0.875+0.8485)/5=0.8523

And their loss during training were:

Model A: 0.0193 0.0036 0.0049 0.0006 0.0060

Model D: 0.0056 0.0015 0.0071 0.0102 0.0063

Model G: 0.0025 0.0030 0.0035 0.0019 0.0041

ROC curves for each fold cross-validation for each model: Model A, Model D, Model G, in order

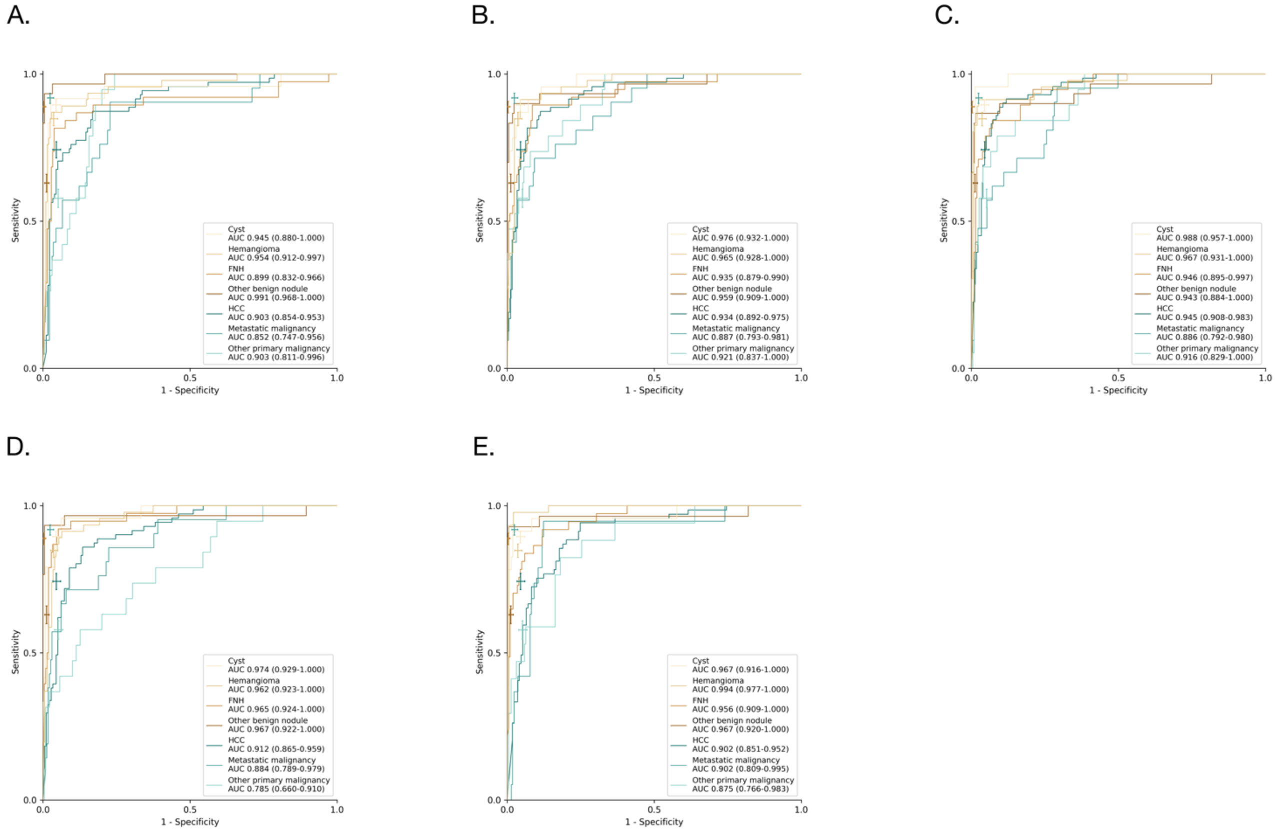
A.
B.
C.
D.
E.
Sensitivity
1 - Specificity
0.0
0.5
1.0
Cyst
AUC 0.945 (0.880-1.000)
Hemangioma
AUC 0.954 (0.912-0.997)
FNH
AUC 0.899 (0.832-0.966)
Other benign nodule
AUC 0.991 (0.968-1.000)
HCC
AUC 0.903 (0.854-0.953)
Metastatic malignancy
AUC 0.852 (0.747-0.956)
Other primary malignancy
AUC 0.903 (0.811-0.996)
Cyst
AUC 0.976 (0.932-1.000)
Hemangioma
AUC 0.965 (0.928-1.000)
FNH
AUC 0.935 (0.879-0.990)
Other benign nodule
AUC 0.959 (0.909-1.000)
HCC
AUC 0.934 (0.892-0.975)
Metastatic malignancy
AUC 0.887 (0.793-0.981)
Other primary malignancy
AUC 0.921 (0.837-1.000)
Cyst
AUC 0.988 (0.957-1.000)
Hemangioma
AUC 0.967 (0.931-1.000)
FNH
AUC 0.946 (0.895-0.997)
Other benign nodule
AUC 0.943 (0.884-1.000)
HCC
AUC 0.945 (0.908-0.983)
Metastatic malignancy
AUC 0.886 (0.792-0.980)
Other primary malignancy
AUC 0.916 (0.829-1.000)
Cyst
AUC 0.974 (0.929-1.000)
Hemangioma
AUC 0.962 (0.923-1.000)
FNH
AUC 0.965 (0.924-1.000)
Other benign nodule
AUC 0.967 (0.922-1.000)
HCC
AUC 0.912 (0.865-0.959)
Metastatic malignancy
AUC 0.884 (0.789-0.979)
Other primary malignancy
AUC 0.785 (0.660-0.910)
Cyst
AUC 0.967 (0.916-1.000)
Hemangioma
AUC 0.994 (0.977-1.000)
FNH
AUC 0.956 (0.909-1.000)
Other benign nodule
AUC 0.967 (0.920-1.000)
HCC
AUC 0.902 (0.851-0.952)
Metastatic malignancy
AUC 0.902 (0.809-0.995)
Other primary malignancy
AUC 0.875 (0.766-0.983)

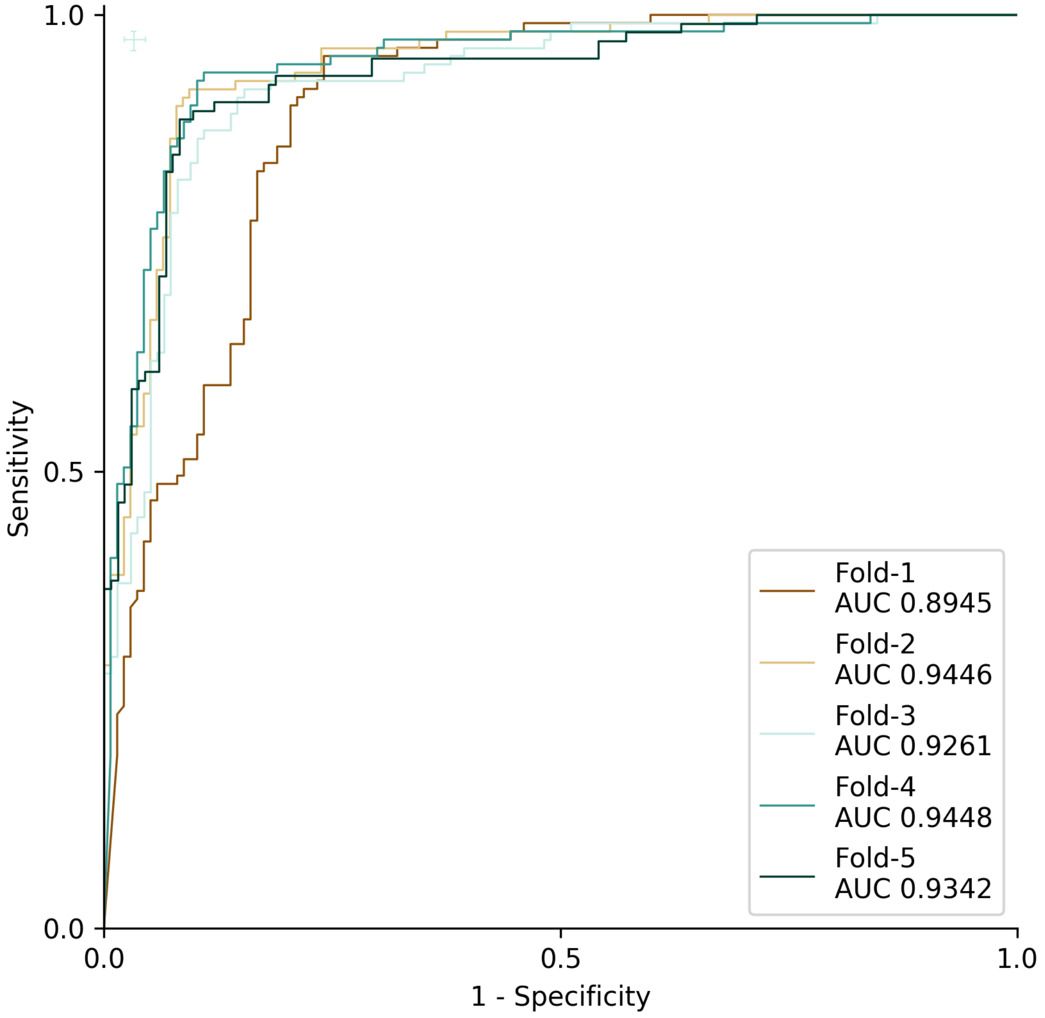

1.0
0.5
0.0
Sensitivity
0.0
0.5
1.0
1 - Specificity
Fold-1
AUC 0.8945
Fold-2
AUC 0.9446
Fold-3
AUC 0.9261
Fold-4
AUC 0.9448
Fold-5
AUC 0.9342

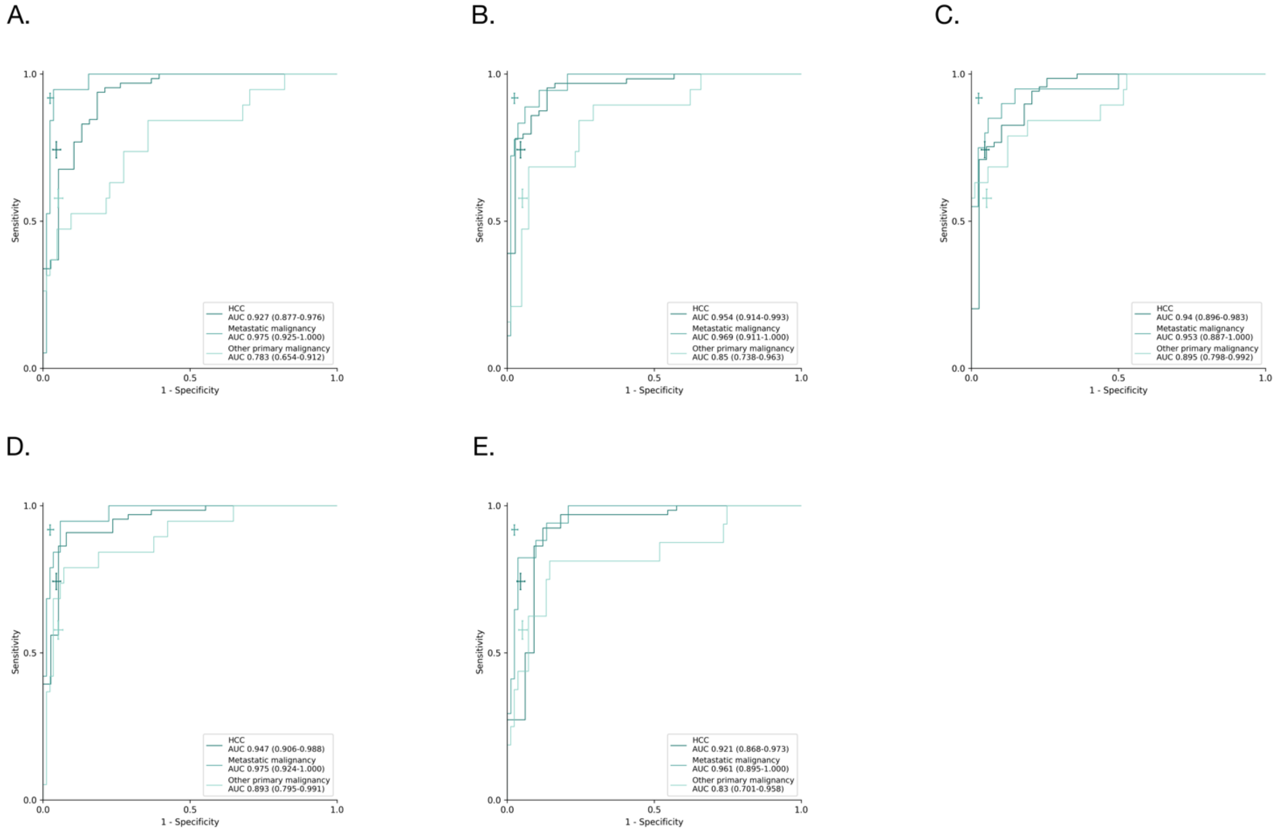
A.
Sensitivity
1 - Specificity
HCC
AUC 0.927 (0.877-0.976)
Metastatic malignancy
AUC 0.975 (0.925-1.000)
Other primary malignancy
AUC 0.783 (0.654-0.912)
B.
Sensitivity
1 - Specificity
HCC
AUC 0.954 (0.914-0.993)
Metastatic malignancy
AUC 0.969 (0.911-1.000)
Other primary malignancy
AUC 0.85 (0.738-0.963)
C.
Sensitivity
1 - Specificity
HCC
AUC 0.94 (0.896-0.983)
Metastatic malignancy
AUC 0.953 (0.887-1.000)
Other primary malignancy
AUC 0.895 (0.798-0.992)
D.
Sensitivity
1 - Specificity
HCC
AUC 0.947 (0.906-0.988)
Metastatic malignancy
AUC 0.975 (0.924-1.000)
Other primary malignancy
AUC 0.893 (0.795-0.991)
E.
Sensitivity
1 - Specificity
HCC
AUC 0.921 (0.868-0.973)
Metastatic malignancy
AUC 0.961 (0.895-1.000)
Other primary malignancy
AUC 0.83 (0.701-0.958)
